# Cytotoxicity of human antibodies targeting the circumsporozoite protein is amplified by 3D substrate and correlates with protection

**DOI:** 10.1016/j.celrep.2023.112681

**Published:** 2023-06-28

**Authors:** Manuela C. Aguirre-Botero, Lawrence T. Wang, Pauline Formaglio, Eduardo Aliprandini, Jean-Michel Thiberge, Arne Schön, Yevel Flores-Garcia, Shamika Mathis-Torres, Barbara J. Flynn, Lais da Silva Pereira, Yann Le Duff, Mathew Hurley, Adéla Nacer, Paul W. Bowyer, Fidel Zavala, Azza H. Idris, Joseph R. Francica, Robert A. Seder, Rogerio Amino

**Affiliations:** 1 Institut Pasteur, Université Paris Cité, Malaria Infection and Immunity, BioSPC, F-75015, Paris, France; 2 Vaccine Research Center, National Institute of Allergy and Infectious Diseases, National Institutes of Health, Bethesda, Maryland, USA; 3 Department of Biology, Johns Hopkins University, Baltimore, MD, 21218, USA; 4 Department of Molecular Microbiology and Immunology, Malaria Research Institute, Johns Hopkins Bloomberg School of Public Health, Baltimore, MD, 21205, USA; 5 Centre for Aids Reagents, National Institute for Biological Standards and Control (NIBSC), Medicines and Healthcare products Regulatory Agency (MHRA), Blanche Lane, South Mimms, Potters Bar, EN6 3QG, UK; 6 Division of Bacteriology, National Institute for Biological Standards and Control (NIBSC), Medicines and Healthcare products Regulatory Agency (MHRA), Blanche Lane, South Mimms, Potters Bar, EN6 3QG, UK; 7 Present address: Affinity Reagents Team, Biologics Engineering, AstraZeneca, Cambridge, UK; 8 Present address: Vaccines and Immune Therapies, BioPharmaceuticals R&D, AstraZeneca, Gaithersburg, MD, USA; 9 Present address: Faculty of Medicine and Health Sciences, University of Buckingham, Apollo Buckingham Health Science Campus, Crewe, CW1 5DU, UK.; 10 Present address: Science, Research and Innovation Group, Medicines and Healthcare products Regulatory Agency (MHRA), Blanche Lane, South Mimms, Potters Bar, EN6 3QG, UK.; 11 These authors contributed equally; 12 Lead contact

## Abstract

Human monoclonal antibodies (hmAbs) targeting the *Plasmodium falciparum* circumsporozoite protein (PfCSP) on the sporozoite surface are a promising tool for preventing malaria infection. However, their mechanisms of protection remain unclear. Here, using 13 distinctive PfCSP hmAbs, we provide a comprehensive view of how PfCSP hmAbs neutralize sporozoites in host tissues. Sporozoites are most vulnerable to hmAb-mediated neutralization in the skin. However, rare but potent hmAbs additionally neutralize sporozoites in the blood and liver. Efficient protection in tissues mainly associates with high-affinity and high-cytotoxicity hmAbs inducing rapid parasite loss-of-fitness in the absence of complement and host cells *in vitro*. A 3D-substrate assay greatly enhances hmAb cytotoxicity and mimics the skin-dependent protection, indicating that the physical stress imposed on motile sporozoites by the skin is crucial for unfolding the protective potential of hmAbs. This functional 3D cytotoxicity assay can thus be useful for downselecting potent anti-PfCSP hmAbs and vaccines.

## In brief

Aguirre-Botero et al. identify the key determinants underlying anti-PfCSP hmAb protective activity. Protection essentially associates with high-cytotoxicity and high-affinity antibodies. Protective hmAbs mainly neutralize sporozoites moving through the skin. Potent effectors additionally neutralize parasites outside the skin. Remarkably, 3D substrate enhances cytotoxicity, mimics skin protection, and improves prediction of protection.

## Graphical Abstract

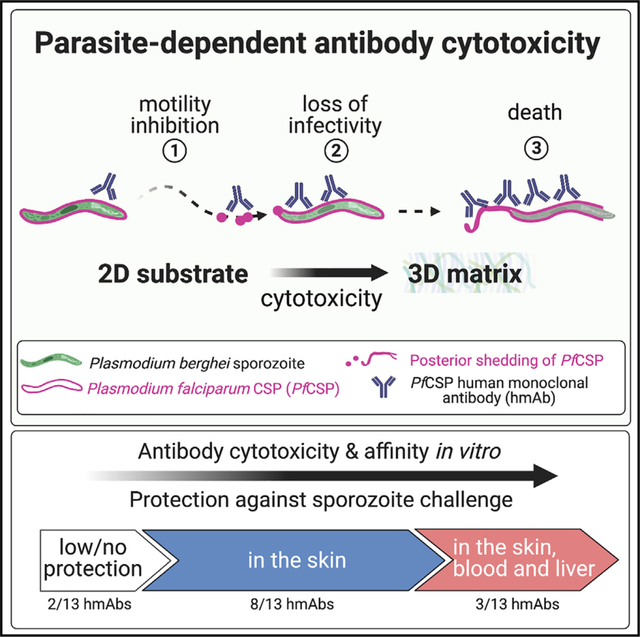

## INTRODUCTION

Malaria is a parasitic mosquito-borne disease caused by *Plasmodium* spp., the deadliest of which is *Plasmodium falciparum* (Pf). Despite significant decreases in incidence and fatalities over the last 15 years, the total number of malaria cases has stayed steady at around 200–250 million annually.^1^ Plasmodial infection starts with the inoculation of sporozoites into the host skin during a mosquito bite,^2^ whereupon these extravascular sporozoites activate motility and enter the circulation by invading cutaneous blood vessels.^3,4^ Circulating sporozoites then rapidly arrest in the liver,^5^ where they extravasate to invade hepatocytes and develop into merozoites, the next stage of the parasite’s life cycle.^6^ After several days these merozoites exit the liver and enter the circulation where they infect erythrocytes, causing malaria symptoms. Given the various cellular barriers sporozoites need to cross and the low number of parasites involved in this asymptomatic liver infection, the pre-erythrocytic phase of *Plasmodium* infection is optimal for immune interventions to prevent malaria.

The circumsporozoite protein (CSP) is the immunodominant sporozoite surface antigen.^7^ The structure of PfCSP comprises a proteolytically processable N-terminal region, a central region composed of repetitive tetrapeptides (1 NPDP, 4 NVDP, and 38 NANP in the 3D7 reference strain) which are the main target of protective antibodies, and a C-terminal region attached by a putative glycosylphosphatidylinositol (GPI) anchor to the sporozoite membrane (Table 1). RTS,S/AS01, the only malaria vaccine recommended for widespread usage in regions with moderate-to-high Pf transmission, is based on a truncated version of PfCSP composed of 19 NANP repeats and the C terminus fused to the hepatitis B surface antigen.^13^ This vaccine elicits high anti-PfCSP antibody titers against the repeat region that are associated with protection,^14,15^ but the precise mechanism by which these antibodies neutralize sporozoites *in vivo* is unclear. Henceforth, neutralization will be employed to characterize any antibody activity leading to a decrease in sporozoite fitness and ultimate ability to infect hepatocytes.

Anti-PfCSP human monoclonal antibodies (hmAbs) isolated from volunteers vaccinated with RTS,S^10^ or attenuated Pf sporozoites,^9,12,16^ as well as from subjects naturally exposed to malaria,^17^ can elicit sterile protection in murine^9,10,12,16,17^ and human^18,19^ sporozoite challenge models. Some studies have reported that neutralization of sporozoites *in vivo* by anti-PfCSP hmAbs correlates with affinity to the KQPADGNPDPNANPN or to NANP 22–24mer peptides.^12,20,21^ However, increased affinity alone does not predict the potency of PfCSP hmAbs.^8,20^ Studies have also demonstrated that the hepatocyte invasion assay, which is the most widely used *in vitro* sporozoite neutralization assay, does not reliably predict hmAb efficacy against intravenous (i.v.) or mosquito bite challenge.^8,9^ Thus, there is still no *in vitro* assay to predict the *in vivo* potency of PfCSP hmAbs. Furthermore, some anti-CSP antibodies are only protective if sporozoites are delivered into the skin^22^ while others are protective against both skin and i.v. injection of sporozoites,^8^ suggesting that CSP antibodies may have different effects on sporozoites depending on the concentration and physiological site of action. Overall, these data highlight the need for a better understanding of the mechanisms underlying protection mediated by PfCSP hmAbs in relevant tissues that could lead to the identification of a robust correlate of humoral protection against sporozoite infection.

## RESULTS

### Skin is the main site of protection, but additional protection in the blood and liver characterizes potent effectors

**Figure 1.**
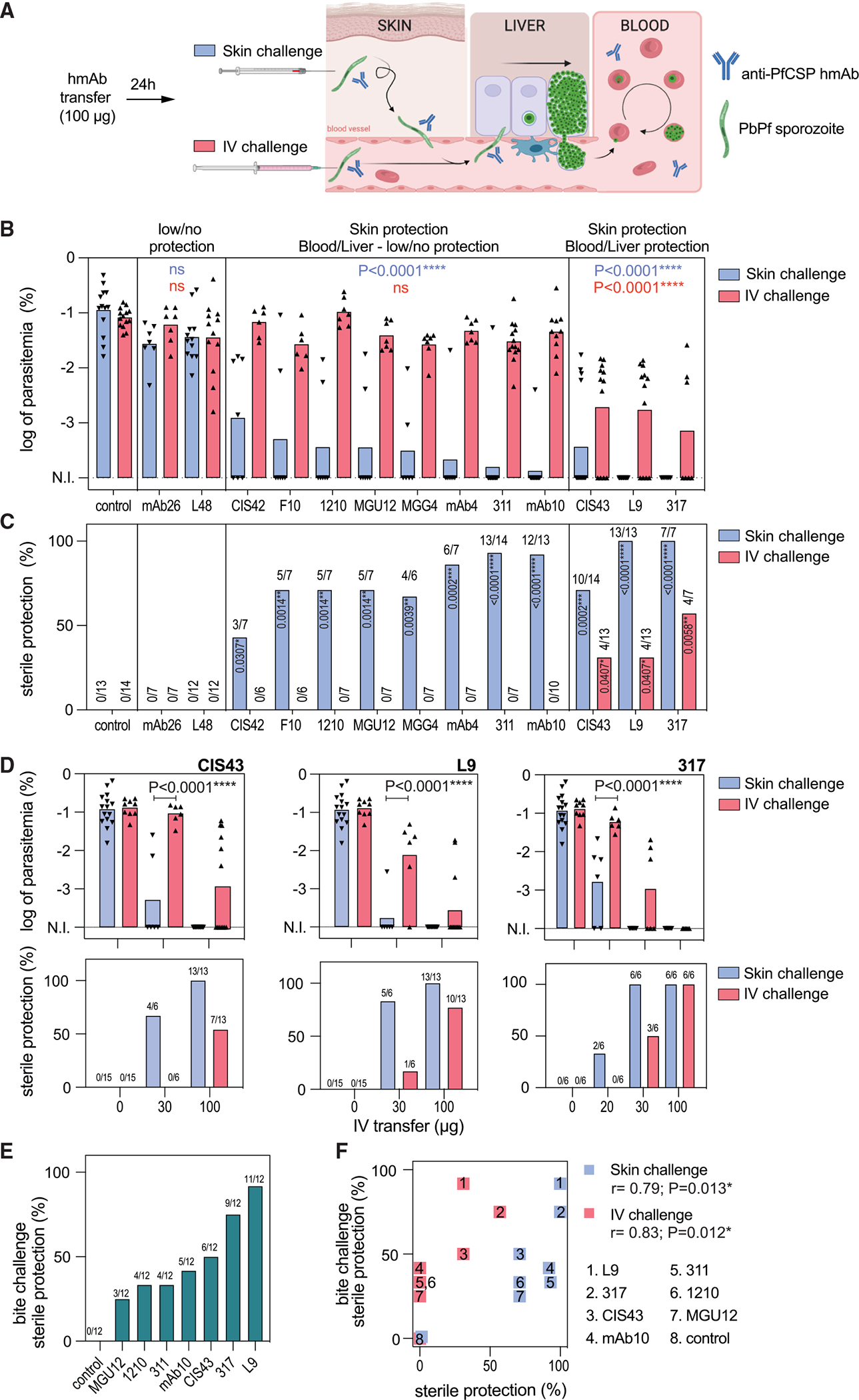
Skin is the major site of sporozoite vulnerability to anti-PfCSP hmAbs, and potent effectors also neutralize sporozoites in the blood and liver (A) Strategy to quantify tissue-dependent protection elicited by anti-PfCSP hmAbs. Mice were transferred with hmAbs and 24 h later microinjected with 5,000 PbPf sporozoites in the footpad (skin challenge, blue) or with 1,000 PbPf sporozoites intravenously (IV challenge, red). Liver infection was estimated by ensuing parasitemia. Cartoon created with BioRender.com. (B and C) (B) Comparison of the log parasitemia at day 5 post challenge and (C) sterile protection between PfCSP hmAb transferred and control groups (number of sterile protected/challenged mice above the bars). For control, L48, 311, mAb10, CIS43, L9, and 317 hmAbs, n = 11–13 mice per group, mice pooled from two independent experiments. For mAb26, CIS42, 1210, MGU12, MGG4, and mAb10 hmAbs, n = 7 mice per group from a single experiment. (D) Titration of protective efficacy of CIS43, L9, and 317, 24 h after i.v. transfer by log parasitemia (top) and sterile protection (bottom) after i.v. or skin challenge; n = 6–13 mice per group pooled from 2–3 independent experiments. For 317 (20 μg), n = 6 mice per group from a single experiment. (E) Sterile protection mediated by i.v. transfer of 300 μg of hmAbs 24 h before mosquito bite challenge. The number of protected/challenged mice is shown on the top of the bars; n = 12 mice per group pooled from two independent experiments. (F) Spearman’s correlation between the sterile protection achieved after skin/i.v. and mosquito bite challenge 24 h after hmAb transfer (blue and red dots, respectively). In (B) and (D), we used one-way ANOVA corrected for multiple comparisons (Holm-Šídák). Groups were compared with respective control; ns (p > 0.05); ****p < 0.0001. In (C), we used Fisher’s exact test. N.I., non-infected.

To dissect the protection mechanism of anti-PfCSP repeat hmAbs in the physiological sites where sporozoites transit, we first established a quantitative method to estimate the protective efficacies of hmAbs in the skin, blood, and liver of mice challenged with transgenic rodent-tropic GFP-expressing *Plasmodium berghei* (Pb) sporozoites, in which the endogenous PbCSP was replaced by PfCSP (PbPf).^8^ Protection was quantified based on (1) theoncavse in parasitemia measured 5 days after sporozoite challenge when parasites are growing exponentially in the blood or (2) the percentage of sterile protection, defined as the absence of parasitemia at 10 days post challenge. Thirteen published anti-PfCSP hmAbs with different binding affinities for the three tetrapeptide epitopes of the PfCSP repeat region (NPDP, NVDP, and NANP) and with different protective potencies were used (Table 1). One day after intraperitoneal transfer of 100 μg of hmAbs, mice were either inoculated with 1,000 PbPf sporozoites in the tail vein (i.v. challenge) or microinjected with 5,000 PbPf sporozoites in the footpad (skin challenge, Figure 1A). These inoculums led to comparable parasitemia in control mice (Figure 1B, control), suggesting that the number of sporozoites ultimately infecting the liver via these different routes was similar. I.v. challenge therefore served to measure hmAb neutralization of sporozoites in the blood and liver (BL), while skin challenge allowed estimation of neutralization in both the skin and BL (Figure 1A).

Following passive transfer and challenge three distinct groups of hmAbs were defined, based on protective efficacy (Figures 1B and 1C). The hmAbs in the first group (mAb26 and L48) were classified as eliciting low to no protection in both skin and BL because they did not significantly decrease mean parasitemia after skin or i.v. challenge when compared with control animals. The hmAbs in the second group (CIS42, F10, 1210, MGU12, MGG4, mAb4, 311, and mAb10) were classified as protective in the skin but elicited low to no protection in the BL because they significantly reduced parasitemia and sterilely protected mice following skin challenge but not after i.v. challenge. The hmAbs in the third group (CIS43, L9, and 317) were classified as protective in both skin and BL because they significantly reduced parasitemia and sterilely protected mice following skin and i.v. challenge. Nevertheless, dose titration of CIS43, L9, and 317 demonstrated that these highly protective hmAbs were also more protective against skin challenge compared with i.v. challenge at lower doses (Figure 1D).

To corroborate the results of skin and i.v. challenge with those obtained via mosquito bite challenge, which resembles natural transmission, mice were challenged with bites of five infected mosquitoes 24 h after i.v. administration of 300 μg of hmAb. This bite challenge study confirmed 317 and L9 as the most potent hmAbs (Figure 1E) and correlated significantly with the skin and i.v. challenge protection data (Figure 1F). The lower sterile protection elicited by the PfCSP hmAbs against mosquito bite challenge despite the transfer of more antibody (300 versus 100 μg) indicated that the bite challenge was more infectious than the skin challenge. This could be due to the higher number of infectious sporozoites injected by mosquito bite versus microsyringe, which was not quantified in this experiment. To address this point, we controlled the number of mosquito bites to result in similar parasitemia in skin and i.v. challenged mice (Figure S1). Following the transfer of 100 μg of hmAb 311, 67% of mice remained uninfected after the mosquito bite challenge versus 93% (p = 0.18) and 0% (p < 0.0001) after the skin and i.v. challenge, respectively (Figure S1). This result confirms that the mosquito bite challenge is more similar to the skin challenge than the i.v. challenge.^22^ It also shows a trend suggesting that sporozoites inoculated by bite are less susceptible to hmAb-mediated neutralization than microinjected sporozoites. This could be due to a shorter transit time in the skin or to an increased infectivity of sporozoites transmitted by the mosquito saliva. Together, these results show that protective PfCSP hmAbs predominantly neutralize sporozoites in the skin and that the most potent hmAbs can additionally neutralize the sporozoites that rapidly enter the blood circulation.

### *In vitro* parasite-dependent cytotoxicity correlates with protection against sporozoites delivered in the skin and in the blood

**Figure 2.**
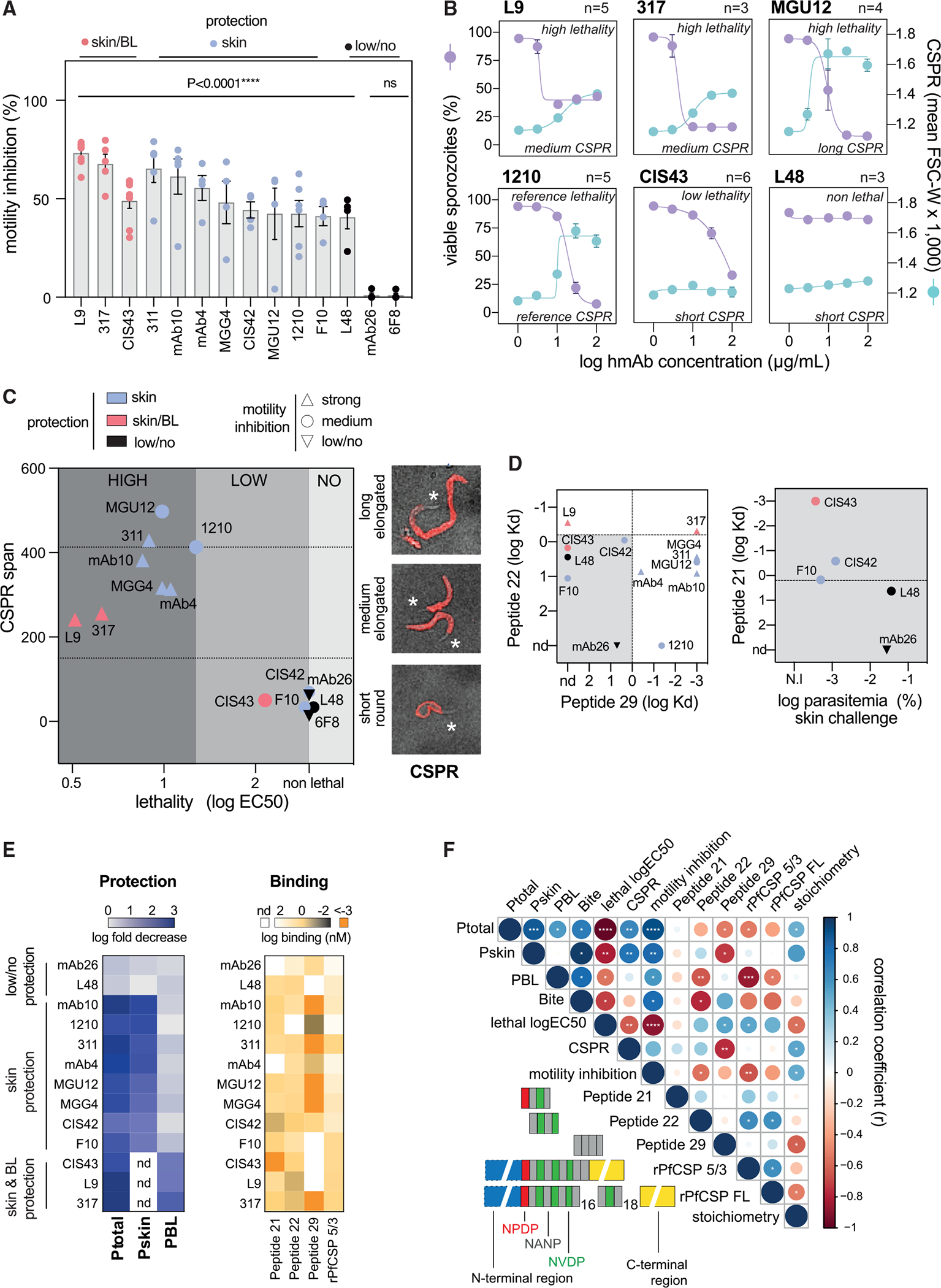
Cytotoxicity *in vitro* very strongly correlates with protection *in vivo* (A) *In vitro* motility inhibition of PbPf sporozoites on a two-dimensional (2D) substrate. Each dot represents the percentual decrease of the averaged speed compared with the PBS/10% FCS control for each independent experiment. Comparison using ANOVA with Holm-Šídák correction (n = 4–6 independent experiments, data are presented as means ± SEM). 6F8 is an isotype control hmAb that binds to the rPfCSP but does not recognize PbPf sporozoites.^34^ (B) Representative plots depicting the distinct *in vitro* lethality (purple) and CSPR (blue) patterns induced in PbPf sporozoites by hmAbs after 45 min of incubation at 37°C (n = 3–6 independent experiments, data are presented as mean ± SEM). (C) Left: distribution of hmAbs according to high/low/no lethality, CSPR span, *in vivo* protection (purple, mainly in the skin; red in the skin and BL; black, no/low protection), and motility inhibition (upward triangle, strong inhibition; circles, medium inhibition; downward triangles, low/no inhibition). Right: representative images of three distinct CSPR phenotypes induced by PfCSP hmAbs. (D) Left: distribution of hmAbs by log avidity to peptides 21 and 22. The hmAbs inducing short-round CSPR with low/no lethality (squares) are delimited by the gray rectangle. Right: avidity to peptide 21 of hmAbs in the gray rectangle against the log parasitemia after the skin challenge. (E) Left: comparison of the fold change in protection after skin challenge (Ptotal), protection in the skin (Pskin = Ptotal − PBL), and protection after i.v. challenge (PBL). nd, not determined. Right: heatmap of log avidity/affinity for peptides 21, 22, and 29, and the truncated rPfCSP5/3. (F) Matrix depicting Spearman’s correlation between various *in vivo* protection quantifications (Ptotal, Pskin, PBL), *in vitro* neutralization activities, and binding parameters for the hmAbs panel. *p < 0.05, **p < 0.01, ***p < 0.001, ****p < 0.0001.

Having classified the protective efficacies of the PfCSP hmAb panel across the three challenge models, we next sought to define the mechanisms by which these hmAbs neutralize sporozoites. Anti-CSP antibodies have been shown to induce the posterior translocation of the CSP surface coat in a thread-like structure (circumsporozoite precipitation reaction [CSPR], FigureS2),^23^ inhibit parasite motility,^24^ and trigger the killing of sporozoites *in vitro* without downstream host effectors, such as the complement and cytotoxic immune cells.^22^ To test whether *in vivo* protection was associated with these *in vitro* primary neutralization phenotypes, we first quantified the inhibitory effect of 10 μg/mL hmAbs on sporozoite motility using a two-dimensional (2D) plastic substrate (Figure 2A). Motility inhibition was assessed by the decrease in the sporozoite speed analyzed between 3 and 5 min of incubation at 37°C and 5% CO_2_. All hmAbs significantly inhibited 2D motility except the non-protective, low-affinity mAb26. Next, we simultaneously quantified the capacity of hmAbs to kill and induce the CSPR in sporozoites in suspension after 45 min of incubation at 37°C by flow cytometry^22^ (Figure S2A). Sporozoite killing was quantified by the loss of cytoplasmic GFP fluorescence and nuclear incorporation of propidium iodide and assessed based on the half-maximal lethal effective concentration of hmAbs (EC_50_). The extent of the CSPR was measured by estimating the length of sporozoites using the forward scatter-width light signal (FSC-W). The CSPR span thus represents how long the parasite stretched after incubation with hmAbs (maximum minus minimum FSC-W). Qualitatively, the CSPR was analyzed by confocal microscopy using a fluorescently labeled antibody (Figure S2B).

The hmAbs displayed variable levels of lethality (high, low, and no) and CSPR phenotypes (short-round, medium-elongated, and long-elongated) (Figures 2B, 2C, and S2B). Given its intermediate potency, hmAb 1210 was selected as an arbitrary reference to delineate killing and CSPR levels (Figure 2C). All hmAbs that elicited a short-round CSPR displayed undetectable (mAb26, L48, F10, CIS42) or low (CIS43) lethality (Figures 2C and S2). These hmAbs presented the weakest binding to peptide 22 (NVDP, log avidity > −0.05 nM) and peptide 29 (NANP, log avidity > 0 nM; Figure 2D, left graph, gray rectangle). In this group, protection against skin challenge was associated with stronger binding to peptide 21 (NPDP, Figure 2D, right graph). Contrarily, all highly lethal hmAbs elicited a medium- or long-elongated CSPR, suggesting that strong binding to peptide 22 (NVDP) and peptide 29 (NANP) is linked to CSP shedding and parasite killing in suspension. The stripping of the CSP coat at the anterior end of dead sporozoites caused by lethal hmAbs that induced a medium-elongated (L9 and 317) or short-round CSPR (CIS43) (Figures 2C and S2B) suggests that exhausting the CSP intracellular storage is unnecessary for uncoating the parasite membrane and killing sporozoites.

To further interrogate the hmAbs protective profiles, using the data from Figure 1B we subdivided their protective effects into different tissues (i.e., skin or BL). To quantify tissue protection, we calculated how strongly hmAbs decreased parasite infection by subtracting the mean log parasitemia of control mice from the mean log parasitemia at day 5 after skin and i.v. challenge in mice that received hmAbs (Figure S3A). One unit of protection thus represents a 10-fold decrease in parasite infection. Total protection (Ptotal) was then defined as the protection elicited by hmAbs in both skin and BL after the skin challenge. Protection in the BL (PBL) was similarly defined as the protection elicited by hmAbs only in the BL following i.v. challenge. Consequently, protection in the skin (Pskin), which represents the impact of sporozoite passage through the skin in protection, was estimated by subtracting the protection measured after i.v. challenge from the protection after skin challenge (Pskin = Ptotal − PBL) (Figures 2E and S3). CIS43, L9, and 317 were excluded from Pskin quantification (Figure 2E, not determined [nd]) because their PBL was too high, impeding an accurate calculation of Pskin.

Having defined the tissue-specific protection (Ptotal, Pskin, PBL) mediated by PfCSP hmAbs, we next correlated these three *in vivo* protection parameters with each other and with the *in vitro* neutralization and binding phenotypes of each hmAb (Figures 2D–2F and S4). Positive and negative correlations with coefficients I between ± (0.51 and 0.70), ± (0.71 and 0.90), and ± (0.91 and 1.0) were respectively considered as moderate, strong, and very strong. *In vitro* activities may show negative correlations with protection when they are measured on the basis of hmAb concentration, such as in the lethality assay (log EC_50_). In this case, potent effectors with lower values of EC_50_ will associate with higher protection.

Ptotal most strongly correlated with lethality (r = −0.97, p < 0.0001), motility inhibition (r = 0.91, p < 0.0001), and Pskin (r = 0.88, p = 0.0004). These data suggest that the hmAbs that most potently slowed or killed sporozoites *in vitro* were most protective against *in vivo* skin challenge. There were some notable outliers to this paradigm. For instance, L48 inhibited sporozoite motility comparably with the other hmAbs (Figure 2A) but was non-protective against skin challenge (Figure 1), while F10 and CIS42 were non-lethal to sporozoites in suspension (Figure S2) but protective against skin challenge (Figure 1). Pskin strongly correlated with CSPR (r = 0.76, p = 0.005), motility inhibition (r = 0.81, p = 0.002), and lethality (r = −0.81, p = 0.002). PBL most strongly correlated with affinity for the junctional region of the central repetitive domain of PfCSP, which contains the three types of tetrapeptides NPDP, NVDP, and NANP (rPfCSP_5/3_, r = −0.85, p = 0.0002), and moderately with motility inhibition (r = 0.57, p = 0.01) and lethality (r = −0.55, p = 0.02). In general, protective hmAbs displayed high affinity to regions including any of these three tetrapeptides as well as high motility inhibition and killing of sporozoites.

The parasite-dependent cytotoxicity of anti-CSP antibodies was previously defined based on the direct killing of sporozoites associated with parasite motility and cell-wounding activities.^22^ It is likely that the motility inhibition and killing of sporozoites respectively measured after 3–5 min and 45 min of incubation with hmAbs are temporally distinct stages of the same loss-of-fitness process triggered by cytotoxic PfCSP hmAbs. Accordingly, these two activities displayed the strongest correlation among the 36 correlative pairs comparing the nine *in vitro* activities tested in Figure 2F (r = −0.88, p < 0.0001, Figure S5A). Lethality and motility inhibition additionally displayed an almost perfect association between their 12 correlation coefficients, indicating that their relationship with all other *in vivo* and *in vitro* parameters is nearly identical (Figure S5B, r = −0.98, p < 0.0001). Henceforth, we use the term cytotoxicity to characterize the neutralizing activity of antibodies targeting the CSP leading to a decrease in sporozoite motility and culminating in parasite death.

Collectively, the consistent correlation of both motility inhibition and sporozoite-killing activities with Ptotal, Pskin, PBL, and sterile protection against mosquito bite challenge indicates that PfCSP hmAbs largely protect against sporozoite challenge by exerting a cytotoxic effect on parasites. Accordingly, the two most protective hmAbs are the two best cytotoxic effectors. Importantly, protection after skin challenge almost perfectly correlated with *in vitro* sporozoite killing by hmAbs, indicating that this assay could be deployed to evaluate and predict *in vivo* efficacy of most anti-PfCSP antibodies. However, some hmAbs with low cytotoxicity but eliciting high protection, such as CIS43, will not be identified by this assay.

### Cutaneous cytotoxicity correlates with protection but cannot completely block sporozoites in the skin

**Figure 3.**
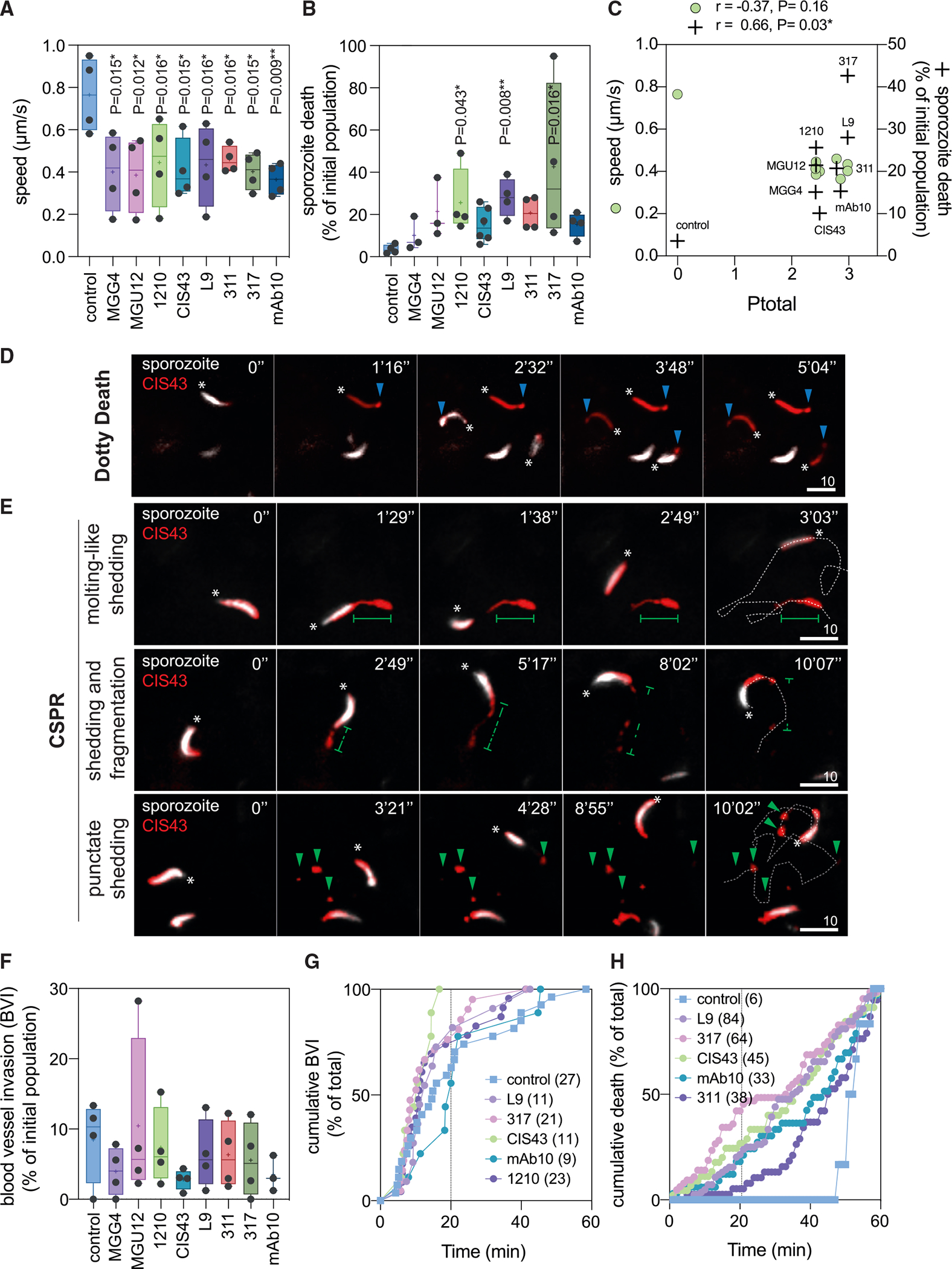
PfCSP hmAbs inhibit motility, induce CSPR, and kill sporozoites in the skin but do not fully prevent sporozoites from invading blood vessels Mice were transferred with 90 μg of unlabeled and 10 μg of Alexa Fluor 647-labeled hmAbs. Twenty-four hours later, PbPf sporozoites were microinjected in the ear skin and imaged for 1 h. Data are from 3–6 independent experiments. (A) Analysis of speed from 7 to 9 min post injection. Comparison with the control mean using ANOVA with Holm-Šídák correction. *p < 0.05, **p < 0.01. (B) Percentage of death during 1 h of observation. Comparison with control mean rank using Kruskal-Wallis test. *p < 0.05, **p < 0.01. (C) Spearman’s correlation between mean of motility inhibition and lethality, and Ptotal. (D and E) Representative time-lapse images of sporozoites (SPZ, white) in the skin of mice transferred with CIS43 (red). Asterisks depict the sporozoite anterior pole. Scale bars, 10 μm. (D) Cytotoxic “dotty death” phenotype characterized by the loss of GFP fluorescence (white) and the formation of a dotty structure in the posterior pole of sporozoites (blue arrowheads). (E) Exemplary and recurrent patterns of CSPR (molting-like shedding, shedding and fragmentation, and punctate shedding). Green lines delineate the “tail” of PfCSP shed from the posterior pole of sporozoites; green arrowheads depict punctate PfCSP shed by the sporozoites; dotted white lines delineate the sporozoite trajectory. (F) Percentage of blood vessel invasion (BVI) normalized by the initial number of sporozoites in 1 h of observation. (G) Normalized cumulative kinetics of BVI in 1 h. Number of invasions is shown in parentheses. (H) Normalized cumulative kinetics of sporozoite death. The total number of dead sporozoites is shown in parentheses. In (A), (B), and (F), box plots show the 25th to 75th percentiles, median, and mean (“+”), and the whiskers extend to the minimum and maximum values.

To determine whether the best *in vitro* correlates of Ptotal, motility inhibition, and sporozoite killing were directly linked to sporozoite arrest in the dermis, we dynamically measured these cytotoxic neutralization phenotypes for a subset of protective hmAbs (MGG4, MGU12, 1210, CIS43, L9, 311, 317, and mAb10) on PbPf sporozoites in the skin of mice using high-speed spinning-disk confocal microscopy.^25^ Twenty-four hours after passive transfer of 100 μg of PfCSP hmAb (90 μg of unlabeled and 10 μg of Alexa Fluor 647 labeled), GFP-expressing sporozoites were microinjected in the ear and observed over 1 h. All eight hmAbs significantly inhibited sporozoite motility between 7 and 9 min post challenge (Figure 3A) and increased sporozoite death (3- to 12-fold, Figure 3B). However, only *in vivo* sporozoite killing moderately correlated with Ptotal (Figure 3C, r = 0.66, p = 0.03). Consistent with the previously reported “dotty death” phenotype,^22^ sporozoite killing was frequently associated with the formation of a dotty structure at the parasite’s posterior end (Figure 3D and Video S1).

Interestingly, introducing sporozoites into the cutaneous environment markedly modified the cytotoxicity patterns observed. For instance, CIS43, despite having low lethality *in vitro* (Figure 2B), killed sporozoites *in vivo* similarly to the highly lethal mAb10 (Figure 3B). Furthermore, CIS43 induced a short-round CSPR *in vitro* (Figure S2B) but elicited at least three distinct CSPR patterns *in vivo*. The first pattern was the classical elongated CSPR,^26^ where sporozoites posteriorly shed PfCSP during forward motility in a “molting-like shedding” phenotype (Figure 3E, top panels). The second pattern started with the aforementioned molting-like shedding followed by fragmentation and disappearance of the CSPR fragments (Figure 3E, middle panels). This “shedding and fragmentation” pattern followed by clearance of the CSPR fragments suggested that this phenotype is associated with cellular interactions, e.g., during sporozoite migration through skin cells.^27^ The third and most observed pattern was characterized by the “punctate shedding” of CSPR fragments from the sporozoite’s posterior end during locomotion (Figure 3E, bottom panels). These phenotypes of dotty death and CSPR were also observed with the other seven hmAbs. Notably, many sporozoites locomoted in the dermis without detectable shedding of PfCSP (Video S1).

Despite inhibiting motility, inducing CSPR, and killing sporozoites in the skin (Figures 3A–3E), hmAbs that were protective against skin challenge but not protective against i.v. challenge (1210, MGU12, MGG4, 311, and mAb10; Figure 1) did not significantly prevent sporozoites from invading cutaneous blood vessels and entering the circulation (no to 3-fold decrease; Figure 3F). This discrepancy is explained by the observation that most sporozoites, despite the decrease in speed (Figure 3A), invaded blood vessels within 20 min after microinoculation in the presence of hmAbs (Figure 3F), while hmAb-mediated cytotoxic death was mainly observed 20 min after microinoculation (Figure 3G). This temporal dependency, whereby the sporozoites that do not escape the skin within 20 min are killed by the hmAbs, explained why death percentages (Figure 3B) are uncoupled from blood vessel invasion percentages (Figure 3E). Overall, these data show that after the transfer of 100 μg of PfCSP hmAbs, the direct immobilization and killing of parasites by protective antibodies are not the main cause of sporozoite neutralization in the skin, since the decrease in blood vessel invasion could not account for the level of sterile protection elicited *in vivo* after skin challenge. Nevertheless, *in vivo* killing of sporozoites correlated with Ptotal, indicating once more that hmAb cytotoxicity is a robust proxy of protection and likely underlying the loss of sporozoite infectivity following migration through the skin.

### 3D substrate amplifies and reveals the commonness of hmAb cytotoxicity

**Figure 4.**
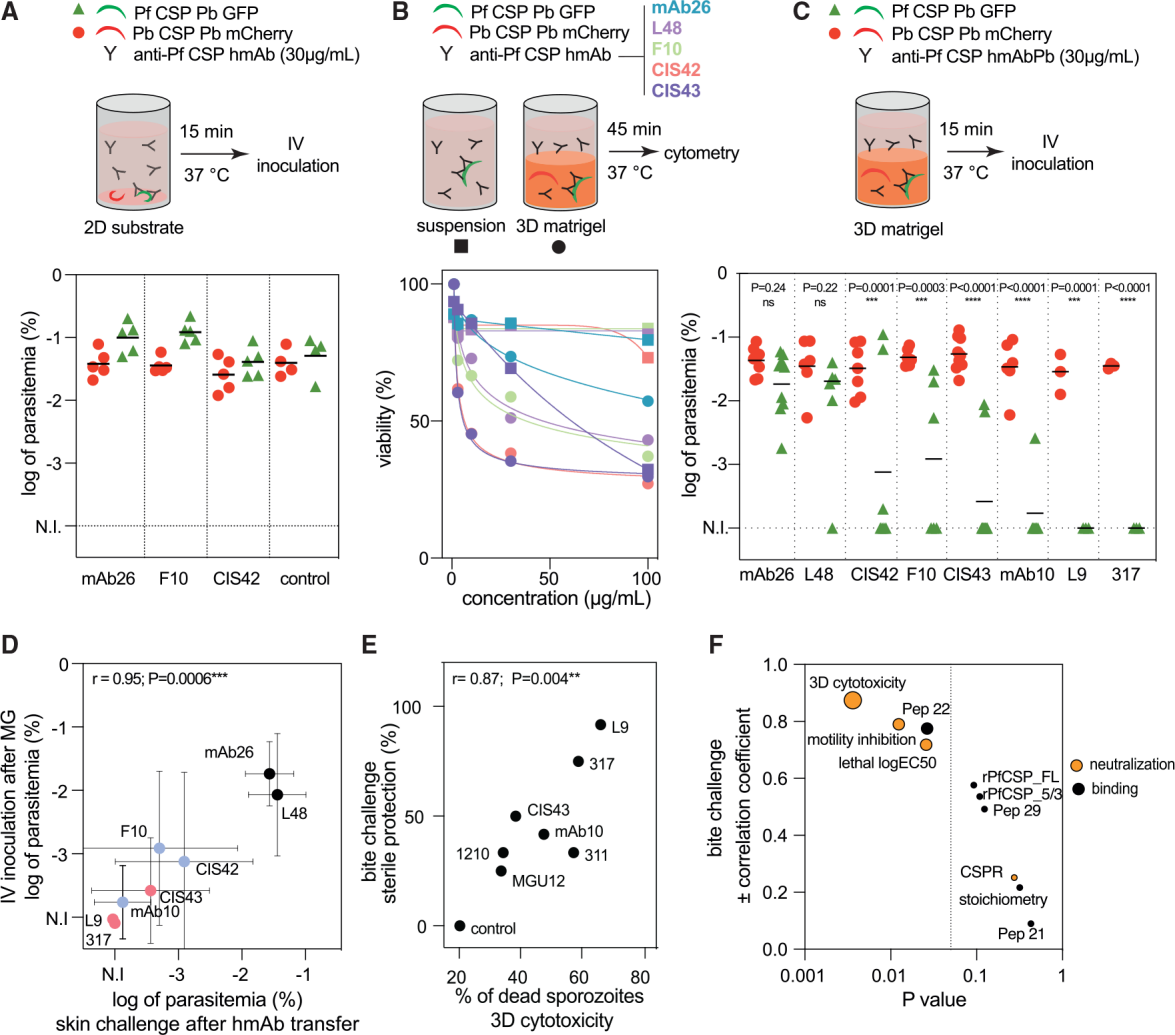
A 3D Matrigel assay recapitulates hmAb cytotoxicity in the skin and strongly correlates with protection after skin and mosquito bite challenge (A) Top: scheme depicting preincubation of a mixture of PbPf sporozoites expressing PfCSP/GFP (green triangles) and Pb sporozoites expressing PbCSP/mCherry (red circles) with 30 μg/mL hmAbs for 15 min at 37°C on a 2D substrate prior to i.v. challenge. Bottom: log parasitemia 5 days after i.v. challenge (n = 4–5 mice from a single experiment). (B) Top: scheme depicting preincubation of sporozoites with hmAbs in suspension (squares) or in 80% Matrigel (3D Matrigel, circles) for 45 min at 37°C prior to flow-cytometry analysis. Bottom: viability of sporozoites in suspension or 3D Matrigel (n = 3 independent experiments, data are presented as the average). (C) Top: scheme for preincubating sporozoite mixture with 30 μg/mL hmAbs for 15 min at 37°C on a 3D-substrate prior to i.v. challenge. Bottom: log parasitemia 5 days after i.v. challenge (n = 6–9 mice pooled from two independent experiments; for L9 and 317, n = 3 mice from a single experiment). (D) Spearman’s correlation between sporozoite infectivity (log parasitemia at day 5) after passage through the skin (x axis) or Matrigel (y axis). Average ± SD. (E) Spearman’s correlation between sterile protection against mosquito bite challenge (Figure 2D) and 3D cytotoxicity of hmAbs (percentage of sporozoite death at 3 μg/mL). (F) Graph depicting 3D cytotoxicity as the best correlate of sterile protection after bite challenge among various binding and *in vitro* neutralizing activities.

Given the discrepant observations that hmAbs are highly protective against skin challenge (Figure 1) despite allowing many sporozoites to invade cutaneous blood vessels (Figures 3E and 3F), we hypothesized that the sporozoites that successfully enter the circulation might have reduced abilities to infect hepatocytes after being exposed to hmAbs in the skin. To test this hypothesis, before being inoculated i.v. into mice, sporozoites were preincubated for 15 min with or without hmAbs in suspension or on 2D substrate, which respectively reproduce the *in vitro* lethality and motility assays (Figures 2A and 2B). Preincubation with two non-lethal hmAbs, the non-protective mAb26 and the skin-protective F10, did not decrease sporozoite infectivity (Figure S6A). Using an internal parasite load control, we repeated the 2D experiment using a mixture of PfCSP hmAb susceptible GFP^+^ PbPf and non-susceptible mCherry^+^ Pb sporozoites. As before, skin-protective and non-lethal hmAbs F10 and CIS42 did not decrease sporozoite infectivity (Figure 4A). These data indicate that the liquid or 2D-substrate environment used in these experiments, which respectively approximate the conditions sporozoites encounter in the blood or when locomoting on vascular endothelial cells, did not recapitulate the skin-dependent loss of sporozoite infectivity elicited by PfCSP hmAbs.

To extend these studies, we developed an assay to quantify hmAb neutralizing activity on sporozoites in 3D Matrigel, which mimics the cutaneous milieu better than the liquid or 2D conditions used before. Remarkably, all tested hmAbs displayed enhanced levels of sporozoite killing in 3D Matrigel. The hmAbs with no lethality (mAb26, L48, F10, CIS42) or low lethality (CIS43) toward sporozoites in suspension (Figure 2C) had much higher cytotoxicity in this 3D assay (Figures 4B, S6B, and S6C). These data indicate that the physical stress inflicted on sporozoites migrating through a 3D substrate greatly enhances hmAb cytotoxicity. Indeed, CIS43 displayed considerable killing of sporozoites in the dermis (Figure 3B) despite being poorly cytotoxic against sporozoites in suspension (Figure 2B). Together, these data indicate that the 3D environment of the skin is a critical physical barrier that amplifies sporozoite neutralization by anti-PfCSP hmAbs.

### 3D cytotoxicity mechanistically mimics skin-dependent protection and is the best correlate of protection against mosquito bite challenge

To test whether short *in vitro* preincubation with hmAbs in 3D substrate reduces sporozoite infectivity *in vivo*, a mixture of GFP^+^ PbPf sporozoites and mCherry^+^ Pb sporozoites was incubated for 15 min in Matrigel with eight PfCSP hmAbs (mAb26, L48, CIS42, F10, CIS43, mAb10, L9, and 317) before being resuspended in PBS and inoculated i.v. into mice. Remarkably, the hmAb protection profiles in this experiment (Figure 4C) accurately recapitulated those from the skin challenge (Figure 2B). Parasitemia in mice inoculated i.v. with PbPf sporozoites preincubated with hmAbs in 3D practically mirrored parasitemia in mice after skin challenge (r = 0.95, p = 0.0006; Figure 4D), further indicating that sporozoite neutralization in Matrigel closely mimics neutralization after parasite migration through the dermis. Direct killing of sporozoites in the 3D cytotoxicity assay could not completely explain *in vivo* protection, since 30%–70% of sporozoites were still viable after short preincubation with hmAbs in Matrigel (Figure S6D). These results indicate that migration through 3D substrates (e.g., Matrigel and skin) in the presence of hmAbs rapidly reduces sporozoite infectivity (Figure 4C), ultimately leading to their cytotoxic death (Figures 4B and S4D). Furthermore, these data resolve the aforementioned discrepancy of several sporozoites being observed to invade cutaneous blood vessels in the presence of hmAbs (Figure 3F) by elucidating that sporozoites which reach the circulation after being shortly exposed to protective hmAbs in the skin or in Matrigel display impaired infectivity. Most importantly, sporozoite killing in the 3D cytotoxicity assay strongly correlated with sterile protection elicited by hmAbs against mosquito bite challenge (r = 0.87, p = 0.004; Figure 4E), making the 3D cytotoxicity assay the best *in vitro* assay among the ten assays tested to correlate with protection against sporozoite infection initiated by mosquito transmission (Figure 4F).

## DISCUSSION

Canonical antibody cytotoxicity is a key mechanism to kill and eliminate target cells by activating host factors, such as the complement cascade and immune cells.^28^ CSP-specific antibody cytotoxicity, however, depends primarily on parasite factors associated with sporozoite progression in the skin, such as the activation of its actomyosin motor and the secretion of molecules involved in the membrane wounding and traversal of host cells.^22^ Accordingly, previous studies have identified the skin as the principal site in which sporozoites are neutralized by the cytotoxic mAbs 3D11 and J6, targeting the repeats of the Pb and *Plasmodium yoelli* CSP, respectively.^4,22,29^ However, whether cytotoxicity and cutaneous sporozoite neutralization are general traits of protective anti-CSP repeats antibodies is uncertain. Using a large panel of 13 PfCSP hmAbs, we show *in vitro* that all tested anti-repeats hmAbs are cytotoxic in 3D substrate but not in a liquid environment, displaying variable levels of sporozoite killing. Out of the 13 hmAbs, the two effectors with low affinity and low cytotoxicity did not protect mice against sporozoite infection. The remaining 11 hmAbs mainly neutralized sporozoites during cutaneous migration and independently of the preferred targeted PfCSP tetrapeptide (Figures 1 and 4). Cytotoxic neutralization of sporozoites in the skin thus seems to be a common attribute of protective antibodies despite differences in the primary sequences of targeted epitopes in the PfCSP and among the species-specific CSP repeats.

In addition, our data show that PfCSP hmAb parasite-dependent cytotoxicity is amplified by the 3D environment of the host skin. The protective property of this 3D substrate is mimicked by Matrigel, which greatly enhances hmAb cytotoxicity, leading firstly to a rapid loss of sporozoite infectivity and latterly to increased parasite death (Figures 3B and 4). Given that sporozoites locomote using a substrate-dependent motility^30^ and cytotoxic killing is linked to the activation of its motor,^22^ antibody cytotoxicity is potentially augmented by the increased shear stress imposed by the 3D substrate on the whole body of motile sporozoites. Accordingly, the short exposition of activated sporozoites to cytotoxic PfCSP hmAbs on 2D substrate or in liquid, which exerts only partial or low physical stress on activated parasites, does not decrease sporozoite infectiousness (Figures 4 and S6). The emergence of skin as an important physical barrier and harmful obstacle for sporozoite progression is supported by recent evidence showing that mutant sporozoites lacking the structural proteoncavingvin display increased disintegration while locomoting in the skin.^31^ The enhanced vulnerability of sporozoites to cytotoxic CSP-specific antibodies in the host skin can be thus explained by the combination of primary parasite and secondary host factors—sporozoite motility and cell traversal, and the host skin 3D environment—both essential for cutaneous migration and optimal hmAb cytotoxicity. Sporozoite neutralization by CSP-specific antibodies in the skin can thus be described as a progressive cytotoxic process starting with the slowing down, followed by the loss of liver infectivity and immobilization, and culminating in the death of sporozoites.

Although most sporozoites are neutralized in the skin, neutralization in the BL is particularly important for targeting sporozoites that quickly reach the blood circulation and evade the skin-dependent mechanisms of protection. In our experimental setup, 8 out of the 11 PfCSP hmAbs eliciting protection after skin challenge do not significantly protect mice after i.v. challenge. Only CIS43, L9, and 317 significantly neutralize sporozoites in the BL. These three hmAbs were previously identified as the most protective hmAbs against mosquito bite challenge and potent effectors in the liver, decreasing sinusoidal extravasation, hepatocyte traversal, or viability of sporozoites.^8^ However, despite having high neutralizing activity in BL after transfer of 100 μg of hmAbs at concentrations inducing only low or no sporozoite neutralization in BL (10–30 μg), these three hmAbs also mainly neutralize sporozoites in the skin. They share a high affinity to peptides 21 (CIS43), 22 (L9), and 29 (317), and display the highest cytotoxicity (L9 and 317) among the 13 tested hmAbs. Accordingly, PBL correlates with high-affinity binding to the junctional region of the PfCSP central repetitive domain (rPfCSP_5/3_), a conserved region harboring the three NPDP, NVDP, and NANP tetrapeptides present in peptides 21, 22, and 29, as well as with cytotoxicity (Figure 2). The high affinity to rPfCSP_5/3_ could thus simply reflect the strong binding of hmAbs to any of these three tetrapeptides. Additionally, binding to the junctional region could also sterically hinder the access of molecules to the adjoining upstream PfCSP region I/I+, a lysine-rich region that binds heparan sulfate proteoglycans,^32^ contains the putative PfCSP proteolytic processing site,^33^ and is potentially involved in the sporozoite arrest in the liver sinusoids and hepatocyte invasion. Further studies are required to define the mechanism behind why the high-affinity binding of hmAbs to the junctional region neutralizes sporozoites in the BL. Overall, high-level prevention of sporozoite infection seems to require high-affinity, high-cytotoxicity PfCSP hmAbs to efficiently neutralize sporozoites in both the skin and BL (Figures 1 and 2).

Cytotoxicity of PfCSP hmAbs could thus account for most of the sporozoite neutralization in the host tissues. Correlation of 13 neutralizing or binding assays performed *in vitro* or *in vivo* with protection against infection by sporozoites inoculated in the skin, blood, or by mosquito bite confirms the strong association between protection and cytotoxicity. *In vitro* hmAb cytotoxicity measured in sporozoite suspension correlates with protection against skin, blood, and mosquito bite challenge (Figures 2F, 4F, and S4). *In vivo* killing of sporozoites by PfCSP hmAbs equally correlates with total protection against skin challenge (Figure 3C). Notably, 3D cytotoxicity of hmAbs in Matrigel displays the best correlation with protection against mosquito bite challenge among all ten *in vitro* binding and neutralizing activities tested (Figure 4F). Thus, this functional assay that amplifies hmAb cytotoxicity, mechanistically recapitulates skin-dependent neutralization, and strongly correlates with protection could be harnessed to screen the neutralizing activity of CSP-specific hmAbs or serum from vaccinated subjects to hasten the identification of next-generation hmAbs or vaccines for clinical testing.

### Limitations of the study

Although parasite-dependent antibody cytotoxicity strongly and functionally correlated with protection in this model, the contributions of other variables to protection, such as the host-dependent canonical antibody cytotoxicity, or the effective hmAb concentration in the tissues were not evaluated in this study. However, the serum pharmacokinetics of 7 of the 13 hmAbs used in this study were previously studied and not found to be markedly different.^8^ Of note, all 13 hmAbs share the same invariant backbone structure. Additionally, lack of key components of complement- and cell-dependent antibody cytotoxicity, C3 and FcγR, did not affect protection elicited by cytotoxic anti-CSP mAbs in knockout mice,^22^ indicating that even if parasites are being targeted by these effectors they are not significantly contributing to protection. Finally, an important limitation of this study is the use of a rodent malaria model to analyze the activities of hmAbs targeting the PfCSP. This limitation is attenuated by the fact that this model was already successfully employed to select potent effectors, which efficiently protected humans against Pf sporozoite infection. A direct comparison of hmAb neutralizing activities on PbPf and Pf sporozoites could further inform on the limits of using PbPf as a proxy for Pf sporozoites. Ultimately, correlative studies of hmAb neutralizing activities on PbPf with protection efficacy data coming from controlled sporozoite challenge in passively immunized volunteers, as already performed for CIS43^18^ and L9^19^ hmAbs, could validate hmAb cytotoxicity as a predictive proxy for human protection against Pf sporozoite infection.

## STAR★METHODS

### RESOURCE AVAILABILITY

#### Lead contact

Further information and requests for resources and reagents should be directed to and will be fulfilled by the lead contact, Rogerio Amino (roti@pasteur.fr) or Robert A. Seder (rseder@mail.nih.gov).

#### Materials availability

This study did not generate new unique reagents.

#### Data and code availability

- All data reported in this paper will be shared by the lead contact upon request
- This paper does not report original code.
- Any additional information required to reanalyze the data reported in this paper is available from the lead contact upon request.

### EXPERIMENTAL MODEL AND STUDY PARTICIPANT DETAILS

#### Mice

For the bite experiment of Figure 2E, female 6- to 12-week-old C57BL/6 mice were obtained from Charles River Laboratories. All mouse research was performed according to National Institutes of Health (NIH) guidelines for use and care of live animals approved by the institutional animal care and use ethics committees of the Vaccine Research Center (Animal Study Protocol VRC-17–702), and Johns Hopkins University (Approved protocol permit no. MO18H419). For all other experiments, female 4- to 6-week-old RjOrl:SWISS and C57BL/6JRj mice were purchased from Janvier Labs and kept in the animal facility of the CEPIA at the Institut Pasteur in Paris accredited by the French Ministry of Agriculture for performing experiments on rodents. Work on animals was performed in compliance with French and European regulations on care and protection of laboratory animals. All experiments were approved by the Ethics Committee #89 and registered under the references MESR 01324, APAFIS#32422–2021071317049057 v2, APAFIS #32989–2021091516594748 v1.

#### Parasites and mosquitoes

In this study, we used transgenic *Pb* ANKA expressing the full-length *Pf* 3D7 CSP and GFP^8^ or GFP-luciferase,^36^ and *Pb* ANKA expressing mCherry under the control of the HSP70 promotor (Pb mCherry).^35^ *Anopheles stephensi* (SDA 500 strain) mosquitoes were reared at the Center for Production and Infection of Anopheles (CEPIA) at the Institut Pasteur in Paris. Mosquitoes were infected by blood-feeding on parasitized RjOrl:Swiss mice and maintained at 21°C in a humid chamber with sucrose. For all *in vitro* assays and intravital microscopy sporozoites were isolated from the salivary glands of *Anopheles stephensi* 19 to 26 days after the infectious blood meal, filtered on a 35-μm strainer, and kept on ice in 1x Dulbecco’s PBS (PBS) until used. For sporozoite challenge, parasites were obtained from the salivary glands of infected mosquitoes isolated 21–22 days after infection.

#### Cell lines

Expi293 and 293F cells were used from Thermo Fischer Scientific.

### METHOD DETAILS

#### Production of recombinant PfCSP proteins and peptides

Recombinant PfCSP constructs were produced as previously described.^8^ Briefly, sequences corresponding to rPfCSPFL and Junctional region (rPfCSP5/3) were cloned into the same CMV/R-expression vectors with a C-terminal AviTag, HRV3C-processing tag, and a 6X histidine tag (GenScript). rPfCSP constructs were expressed through transient transfection in 293F cells using the Freestyle 293F expression system (Thermo Fisher Scientific) at 37°C, 8% CO_2_ for 6 days, and purified from culture supernatants through polyhistidine-tag affinity chromatography followed by size exclusion chromatography (SEC) on an ÄKTA^™^ Start (GE Healthcare). Monomer-containing fractions were pooled, concentrated, snap frozen, and stored at −80°C. Peptides 21, 22, and 29 were produced by direct synthesis and biotinylated by GenScript.

#### Monoclonal antibodies

The isolation of PfCSP hmAbs used in this study was previously reported.^8–12^ All hmAbs were expressed in IgG1 heavy and light chain plasmids using the ExpiFectamine^™^ 293 Transfection Kit (Thermo Fisher Scientific) and incubated at 37°C, 8% CO_2_ for 6 days. Supernatants were harvested and purified using rProtein A Sepharose Fast Flow resin (GE Healthcare) or protein G chromatography cartridge (Thermo Fisher Scientific). Buffer was exchanged with 1X PBS before being concentrated using Amicon Centrifugal Filters (Millipore). The concentration of purified antibodies was determined using the Nanodrop spectrophotometer (Thermo Fisher Scientific).

#### Biolayer interferometry kinetic binding assay

Antibody binding kinetics were measured using biolayer interferometry (BLI) on an Octet HTX instrument (fortéBio) using streptavidin-capture biosensors (fortéBio), as previously reported.^8^ PfCSP hmAb solutions were plated in black tilted-bottom 384-well microplates (fortéBio); assays were performed with agitation at 30°C. mAb serial concentrations used are as follows: 1.25, 0.625, 0.3125, and 0.15625 μg/mL. Loading of biotinylated peptides 21, peptide 22, and peptide 29 (GenScript) was performed for 300 s, followed by dipping of biosensors into buffer (PBS +1% BSA) for 60s to assess baseline assay drift. Association with whole IgG (serially diluted from 16.67 to 1.04 μM) was done for 300 s, followed by a dissociation step in buffer for 600 s. Background subtraction of nonspecific binding was performed through measurement of association in buffer alone. Data analysis and curve fitting were performed using Octet software, version 7.0. Experimental data were fitted with the binding equations describing a 1:1 analyte-ligand interaction. Global analyses of the complete datasets, assuming binding was reversible (full dissociation), were carried out using nonlinear least-squares fitting allowing a single set of binding parameters to be obtained simultaneously for all concentrations of a given hmAb dilution series.

#### Isothermal titration calorimetry

Isothermal titration calorimetry (ITC) was carried out using a VP-ITC microcalorimeter (Malvern Panalytical), as previously reported.^8^ In brief, the rPfCSP constructs and hmAbs were prepared in 1X PBS, pH 7.4, for all titration experiments. Each antibody solution, prepared at a concentration of ~40 μM (expressed per antigen binding site), was injected in 5 or 7 μL aliquots into the calorimetric cell containing the respective rPfCSP construct. The concentration of rPfCSPFL was ~0.4 μM and Junctional region (rPfCSP5/3) was ~0.8 μM. All titrations were performed at 25°C. The exact concentrations of the reactants in each experiment were determined from the absorbance at 280 nm. The heat evolved upon each injection of antibody was obtained from the integral of the calorimetric signal. The heat associated with binding to the different rPfCSP constructs was obtained by subtracting the heat of dilution from the heat of reaction. The individual heats were plotted against the molar ratio, and the enthalpy change, ΔH, the association constant, Ka (the dissociation constant, Kd = 1/Ka) and the stoichiometry (valency of antigen binding sites), N, were obtained by nonlinear regression of the data to a model that considers the binding to either one or two sets of sites with different binding affinities. Gibbs energy, ΔG, was calculated from the relation ΔG = −RTlnKa, where R is the universal gas constant, (1.987 cal/(K × mol)) and T the absolute temperature in kelvin. The entropy contribution to Gibbs energy, −TΔS, was calculated from the known relation ΔG= ΔH–TΔS. The results were expressed per mole of antigen binding sites and the stoichiometry, N, denotes the number of antigen binding sites per mole of the respective rPfCSP construct.

#### Skin and i.v. challenge

HmAbs (100 μg in 150 μL 1x PBS) were transferred intraperitoneally or intravenously into 4-week-old female C57BL/6JRj mice. 24 h later, mice were challenged with either 1,000 sporozoites injected i.v. into the lateral tail vein using a 30G insulin syringe or 5,000 sporozoites into the footpad skin using a NanoFil syringe (35G, Word Precision Instruments). Parasitemia was followed 4–11 days post-infection by scoring GFP+ events on 250,000–500,000 gated erythrocytes by flow cytometry (CytoFLEX S, Beckman Coulter). Mice without GFP+ events after 10 days post-challenge were considered non-infected.

#### Mosquito bite challenge

Mosquito bite challenge studies using 300 μg/mouse were carried out as previously described (Wang et al., 2020). In brief, 6–8-week-old C57BL/6 female mice (Charles Laboratories) were injected i.v. with 300 μg/mouse of PfCSP hmAbs (blinded and in differing orders per experimental replicate) diluted in PBS in a total volume of 200 μL. Twenty-four hours following hmAb administration, mice were anesthetized with 2% Avertin (Alfa Aesar, Ward Hill, MA), and mosquitoes were allowed to feed on mice for 10 min. Following feeding, mosquito abdomens were inspected to confirm the blood meal. Giemsa staining of blood smears starting on day 4 and up to day 10–12 after exposure to infected mosquito bites was used to check for the presence or absence of parasites. For the bite experiment in Figure S1, 4-week-old female C57BL/6JRj mice were injected intraperitoneally with 100 μg/mouse of the hmAb 311. After 24 h, mice were anesthetized with a mixture of ketamine (50 mg/kg body weight; IMALGENE1000, Boehringer Ingelheim) and xylazine (5 mg/kg body weight, Rompun 2% solution, Bayer). 5 infected mosquitoes/mouse were allowed to bite during 10 min. The animals of each group were rotated between the pots containing the infected mosquitoes every 2 min. Parasitemia was measured from day 4–11 post-infection by scoring GFP+ events on 250,000–500,000 gated erythrocytes by flow cytometry (CytoFLEX S, Beckman Coulter).

#### *In vitro* sporozoite cytotoxicity assay in suspension

PbPf sporozoites were incubated at 37°C for 45 min with different concentrations of hmAbs in the presence of 10% FCS in a final volume of 10 μL containing 10,000–12,000 sporozoites. Samples were next transferred on ice, incubated for 10 min with 5 μg/mL propidium iodide (PI, Invitrogen) and finally diluted 10 times with cold PBS prior to acquisition on a CytoFLEX S flow cytometer (Beckman Coulter).^22^ At least 5,000 events in the sporozoite gate were acquired. Viability was defined as the percentage of GFP+PI− sporozoites to the sum of GFP+PI− and GFP−PI + sporozoites. The Circumsporozoite Precipitation Reaction (CSPR) seen in the presence of anti-CSP antibodies was determined by the intensity of the forward scatter-width signal (FSC-W), which was observed to indicate the length of the sporozoites. CSPR span was determined by subtracting the bottom from the top value of the sigmoidal FSC-W curve. Data were analyzed using the CytExpert 2.0 software (Beckman Coulter).

#### Sporozoite immunofluorescence assay

PbPf sporozoites were treated as described for the *in vitro* cytotoxicity assay in suspension with 100 mg/mL of each monoclonal antibody and 10% FCS/PBS. After 45 min at 37°C, samples were placed on ice, transferred to an 18-well slide (iBidi), fixed with 2% Formaldehyde (Thermo Fisher Scientific) for 20 min at room temperature, blocked and incubated with 4 mg/mL Alexa Fluor ^™^ 647-conjugated goat anti-human IgG (H + L) antibody (Invitrogen) in PBS with 1% BSA for 40 min at room temperature. After washing, wells were filled with mounting medium (iBidi). Images were acquired with an inverted Axio Observer Z.1 microscope (Zeiss) and analyzed using Fiji.^37^

#### *In vitro* gliding motility assay

To assess gliding motility, 5,000 sporozoites were resuspended in 1x PBS containing 5% FCS and 10 μg/mL of hmAb in a final volume of 20 μL. The resulting suspension was transferred to an 18-well slide (iBidi) and centrifuged at 400 g for 3 min at 4°C. The slide was then allowed to equilibrate at 37°C, 5% CO_2_ for 3 min in the incubation chamber (Incubation System S, Zeiss) of an inverted epifluorescence wide-field microscope (AxioObserver Z.1, Zeiss) equipped with a LED illumination system (Colibri2, Zeiss), a CCD camera (AxioCam MR, Zeiss) and controlled by the AxioVision software (version 4.8.2.0, Zeiss). Time-lapse movies were then recorded for 2–4 min at a rate of one image per second with an EC “Plan-Neofluar” 10x/0.3 objective (Zeiss) using a 470 nm LED and a matching filter cube (43HE, Zeiss) to excite and detect GFP and thus visualize sporozoites. Average sporozoite velocity over the first 2 min of the acquisition was determined using the MTrack2 plug-in from Fiji.^38^

#### *In vitro* sporozoite cytotoxicity assay in a 3D environment

PbPf GFP+ and Pb mCherry+ sporozoites were mixed on ice with Corning^®^ Matrigel^®^ matrix (Corning) in a 1:5 ratio. For each condition, the mix was divided into 5 μL reactions containing 10,000 to 12,000 sporozoites. Following polymerization at 37°C for 5 min, one volume of the anti-PfCSP hmAb in 20% FCS/PBS was added (10% FCS final concentration). After 45 min at 37°C, the samples were placed on ice for 10 min to allow depolymerization and diluted 21 times with cold 1x PBS prior to the acquisition on a CytoFLEX S flow cytometer (Beckman Coulter). The number of Pb mCherry+ sporozoites was used to normalize the sporozoite recovery across samples. Viability was defined as the percentage of PbPf GFP+ sporozoites compared to the control Pb mCherry+. Data were analyzed using the CytExpert 2.0 software (Beckman Coulter).

#### Sporozoite challenge after activation in suspension, and on 2D- and 3D-substrates

To assess the infectivity of PbPf sporozoites after activation in suspension, 1,000 sporozoites/mouse were incubated at 37°C for 15 min in 0.5% BSA/DMEM with 30 μg/mL of hmAb. After incubation, samples were diluted in 150 μL 1x PBS and i.v. injected into the lateral tail vein. Similarly, activation on a 2D surface was performed by incubating 2,000 sporozoites/mouse in 0.5% BSA/DMEM and 30 μg/mL hmAb on an 18-well slide (iBidi). After centrifugation at 400 g for 3 min at 4°C and incubation at 37°C for 15 min, the sporozoites were recuperated, re-counted and 1,000 sporozoites per mouse were injected intravenously. Alternatively, the 2D-substrate experiment was performed using Pb mCherry+ sporozoites as control. 2,000 PbPf GFP+ and 2,000 Pb mCherry+ sporozoites were mixed in the same sample and treated as described above. Re-counting was done using Pb mCherry+ sporozoites. To investigate the impact of 3D-substrate in the hmAb cytotoxic activity, PbPf GFP+ and Pb mCherry+ sporozoites were embedded in a 1:5 ratio in Corning^®^ Matrigel^®^ matrix (Corning) on ice. For each condition, the mix was divided into 5 μL reactions containing 1,000 PbPf GFP+ and 1,000 Pb mCherry+ sporozoites/mouse. After polymerization for 5 min at 37°C, 30 μg/mL of the hmAb in 0.5% BSA/PBS were added. The samples were kept for another 15 min at 37°C. After incubation, the samples were placed on ice for 10 min and diluted in 100 μL 1x PBS/mouse. Pb mCherry+ sporozoites served as an internal control to assess sporozoite recovery from the gel and protection using the log of the parasitemia at day 5 post-infection. For all experiments, sporozoites were injected into 4-week-old naive female C57BL/6JRj mice (Janvier Labs). Parasitemia was measured from day 4–11 post-infection by scoring GFP+ events on 250,000–500,000 gated erythrocytes by flow cytometry (CytoFLEX S, Beckman).

#### Intravital imaging in the skin

Intravital imaging in the skin was performed on a spinning-disk confocal system (UltraView ERS, PerkinElmer) controlled by Volocity (PerkinElmer) and composed of 4 Diode Pumped Solid State Lasers (excitation wavelengths: 405 nm, 488 nm, 561 nm and 640 nm), a Yokogawa Confocal Scanner Unit CSU22, a z axis piezoelectric actuator and a Hamamatsu Orca-Flash 4.0 camera mounted on a Axiovert 200 microscope (Zeiss). Z-stacks of 6 plans covering 25 to 30 μm were acquired using an LCI “Plan-Neofluar” 25x/0.8 Imm Korr DIC objective (Zeiss). Two days prior to imaging, the ear pinnae of 4- to 6-week-old female C57BL/6JRj mice were gently epilated with a piece of tape and carefully dabbed with wet gauze to eliminate residual glue. The following day, animals were injected intravenously into the tail vein either with 150 μL of 1x PBS (control) or a mix composed of 90 μg of unlabeled anti PfCSP monoclonal antibody and 10 μg of Alexa Fluor ^™^ 647-coupled antibody diluted in 1x PBS. Around 25 to 30 h following antibody transfer, mice were injected intravenously with 20 μg of Alexa Fluor ^™^ 488-conjugated anti-CD31 antibody (clone 390, Biolegend) to label blood vessels. Animals were then anesthetized with a mixture of ketamine (100 mg/kg body weight, IMALGENE1000, Boehringer Ingelheim) and xylazine (10 mg/kg body weight, Rompun 2%, Bayer), placed under a stereomicroscope and inoculated intradermally with 0.2 μL of PbPf sporozoite suspension, delivered into the dorsal side of the ear pinnae with a microsyringe (NanoFil 10 μL syringe mounted with a 35G bevelled needle, World Precision Instruments). Animals were next transferred onto the microscope stage and inoculation sites were continuously imaged for over an hour, enabling the observation of on average 95 sporozoites per field. During acquisition, mice were kept warm with a heating blanket (Harvard Apparatus) and their anesthesia status was regularly monitored.^25^ Image files were processed and quantified using Fiji.^37^ Sporozoite movements were manually tracked over 2-min movies and mean velocity was determined using the MTrackJ plug-in.^38^ Antibody cytotoxicity *in vivo* was assessed using the gradual loss of parasite fluorescence and subsequent disappearance as a hallmark of parasite death.

### QUANTIFICATION AND STATISTICAL ANALYSIS

Statistical significance was determined with a one-way ANOVA with Holm–Šídák correction for multiple comparisons, two-tailed Fisher’s exact test, or two-tailed unpaired t test with help of GraphPad Prism 9. The Spearman’s rank correlation matrices were done with help of GraphPad Prism 9 and the visualization of the correlations was done using the corrplot package^39^ in R programming language (CRAN, R version 4.0.5).^40^

## Supplementary Material

1

2

## Figures and Tables

**Table 1.** Classification of PfCSP hmAbs used in this study

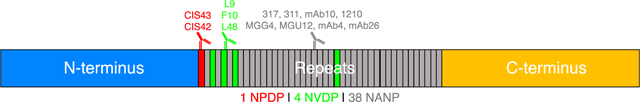												
hmAb	Preferred tetrapeptide	Ig gene family		Apparent avidity (nM)			Two-step binder	Stoichiometry (no. of binding sites)	rPfCSP_FL_ affinity (nM)	rPfCSP_5/3_ affinity (nM)	*In vivo* SPZ neutralization (bite challenge, Wang et al.^8^)	References

		V_H_	V_L_	Pep21	Pep22	Pep29						Wang et al.^8^; Kisalu et al.^9^
CIS43	NPDP	1–3	κ4–1	<0.001	1.5	–	yes	7.2	15	4	high	Wang et al.^8^; Kisalu et al.^9^
CIS42	NPDP	7–4–1	λ2–23	0.26	0.90	2.29	no	11.1	8	66	ND	Kisalu et al.^9^; this study
L9	NVDP	3–33	κ1–5	6.0	0.28	–	yes	11.2	22	3	high	Wang et al.^8^
F10	NVDP	3–33	κ1–5	1.50	11.32	–	no	3.4	14	4	ND	Wang et al.^8^; this study
L48	NVDP	3–23	λ1–47	4.25	2.75	–	yes	11.7	2	66	ND	Wang et al.^8^; this study
317	NANP	3–30/33	κ1–5	1.2	0.49	<0.001	yes	12.2	2	2	high	Wang et al.^8^; Oyen et al.^10^
311	NANP	3–30/33	λ1–40	4.1	3.0	<0.001	yes	15.6	4	2	moderate	Wang et al.^8^; Oyen et al.^10^
mAb10	NANP	3–33	κ1–5	1.6	8.2	<0.001	no	15.2	6	42	moderate	Wang et al.^8^; Kisalu et al.^9^
1210	NANP	3–30/33	κ1–5	2.2	100	0.043	no	12.9	29	600	low	Wang et al.^8^; Imkeller et al.^11^
MGG4	NANP	3–30/33	κ4–1	4.28	2.72	<0.001	no	12.8	12	31	ND	Tan et al.^12^; this study
MGU12	NANP	3–30/33	κ1–5	2.1	3.9	<0.001	no	10.8	7	90	low	Wang et al.^8^; Tan et al.^12^
mAb4	NANP	3–33	κ2D-29	–	7.24	0.39	no	6.1	87	260	ND	Kisalu et al.^9^; this study
mAb26	NANP	3–48	κ1–5	–	–	5.07	no	5.2	1,100	520	ND	Kisalu et al.^9^; this study

Top: graphic of PfCSP depicting the binding profiles of hmAbs used in this study. Schematics depict the N terminus, central repeat region (containing 1 NPDP, 4 NVDP, and 38 NANP tetrapep-tides), and the C terminus of PfCSP from 3D7 reference isolate. Bottom: hmAb name; preferred PfCSP tetrapeptide (NPDP, NVDP, NANP); V_H_ and V_L_ gene families; apparent avidity (nM) for 15mer peptide 21 (NPDPNANPNVDNAN), peptide 22 (NANPNVDPNANPNVD), and peptide 29 (NANPNANPNANPNAN) measured by biolayer interferometry (BLI); two-step binder determined by isothermal titration calorimetry (ITC); stoichiometry (no. of binding sites) of hmAb binding to recombinant full-length PfCSP (rPfCSP_FL_) by ITC; affinity for rPfCSP_FL_ in the first binding event by ITC; affinity for a truncated rPfCSP containing 1 NPDP, 5 NANP, and 3 NVDP (rPfCSP_5/3_) in the first binding event by ITC. Qualitative score of *in vivo* sporozoite neutralization is based on mosquito bite challenge data 72 h after passive transfer of 600 μg hmAb in Wang et al.^8^; high neutralization: >70% sterile protection; moderate neutralization: 31%–69% sterile protection; low neutralization: <30% sterile protection. ND, not determined; “-”, undetectable. Data are representative of 2–3 independent experiments.

**KEY RESOURCES TABLE:** 

REAGENT or RESOURCE	SOURCE	IDENTIFIER

Antibodies		

Anti-human CD31-A Alexa Fluor^®^ 488 (clone 390)	BioLegend	Cat#102414; RRID: AB_493408
Anti-human IgG-Alexa Fluor^®^ 647	Thermo Fisher Scientific	Cat#A-21445; RRID: AB_2535862
Human PfCSP mAbs	Wang et al., 2020^8^ Kisalu et al. 2018^9^	N/A
Control chicken PfCSP mAb (6F8)	Nacer et al., 2022^34^	N/A

Chemicals, peptides, and recombinant proteins		

Recombinant PfCSP constructs with truncated repeat region	Wang et al., 2020^8^	GenBank Accession No. MT891160, MT891178
IMALGENE 1000^®^ (ketamine)	Boehringer Ingelheim	N/A
Rompun 2% solution (xylazine)	Bayer	N/A
Avertin	Alfa Aesar	Cat#A18706
Propidium Iodide	Invitrogen	Cat#P3566
Matrigel^®^ matrix	Corning	Cat#356237
16% Formaldehyde	Thermo Fisher Scientific	Cat#28906

Critical commercial assays		

rProtein A Sepharose	Millipore Sigma	Cat#GE17-1279-03
Expi293 Expression System Kit	Thermo Fisher Scientific	Cat#A14635
FreeStyle 293 Expression System	Thermo Fisher Scientific	Cat#K900001
Protein G chromatography cartridge	Thermo Fisher Scientific	Cat#89927

Experimental models: Cell lines		

Human: Expi293 cells	Thermo Fisher Scientific	Cat#A14527
Human: 293D cells	Thermo Fisher Scientific	Cat#R79007

Experimental models: Organisms/strains		

Mouse: RjOrl:SWISS	Janvier Laboratories	Cat#SN-SWISS-F
Mouse: C57BL/6JRj	Janvier Laboratories	Cat#SC-C57J-F
Mouse: C57BL/6	Charles River Laboratories	Cat#027
Mosquitoes: *Anopheles stephensi* (SDA 500 strain)	CEPIA	N/A
Sporozoite: *P. berghei* expressing PfCSP and GFP	Wang et al., 2020^8^	N/A
Sporozoite: *P. berghei* expressing mCherry	Burda et al., 2015^35^	N/A
Sporozoite: *P. berghei* expressing PfCSP, GFP, and luciferase	Flores-Garcia et al., 2019^36^	N/A

Software and algorithms		

GraphPad Prism 9	GraphPad	http://www.graphpad.com/; RRID:SCR_002798
RStudio	RStudio	https://rstudio.com/; RRID:SCR_000432
R language	CRAN	http://cran.r-project.org/; RRID:SCR_003005
Tidyverse	R package	https://cran.r-project.org/web/packages/tidyverse/index.htm; RRID:SCR_019186
Corrplot	R package	https://github.com/taiyun/corrplot; RRID:SCR_023081
Fiji	Schindelin et al., 2012^37^	https://fiji.sc; RRID:SCR_002285
MTrack2	Fiji Plug-in	https://valelab4.ucsf.edu/~nstuurman/IJplugins/MTrack2.html
MTrackJ	Fiji Plug-in	https://CRAN.R-project.org/package=mdftracks
CytExpert 2.0	Beckman Coulter	https://www.beckman.fr/flow-cytometry/instruments/cytoflex/software; RRID:SCR_017217
Octet Software, version 7.0	FortéBio	http://www.fortebio.com

Other		

CytoFLEX S flow cytometer	Beckman Coulter	N/A
Axio Observer Z.1	Zeiss	N/A
Spinning-disk confocal system Ultra View ERS	Perkin Elmer	N/A
VP-ITC microcalorimeter	Malvern Panalytical	N/A
Octet HTX N/A	ForteBio	N/A

## Highlights

- Skin is the main site of protection, but potent hmAbs also act in the blood and liver
- 3D matrix enhances loss of infectivity and killing of sporozoites by PfCSP hmAbs
- 3D cytotoxicity assay recapitulates PfCSP hmAb skin-dependent protection
- Parasite-dependent hmAb cytotoxicity correlates with protection in tissues
