# Supplementary material for: Cytotoxicity of human antibodies targeting the circumsporozoite protein is amplified by 3D substrate and correlates with protection
Source: Cell Rep. Author manuscript; Available in PMC 2023 Aug 31. (PMC10468621; doi:10.1016/j.celrep.2023.112681)
Supplement: 1 [file NIHMS1920476-supplement-1.pdf]

Cell Reports, Volume *42*

Supplemental information

Cytotoxicity of human antibodies targeting the circumsporozoite protein is amplified by 3D substrate and correlates with protection

Manuela C. Aguirre-Botero, Lawrence T. Wang, Pauline Formaglio, Eduardo Aliprandini, Jean-Michel Thiberge, Arne Schön, Yevel Flores-Garcia, Shamika Mathis-Torres, Barbara J. Flynn, Lais da Silva Pereira, Yann Le Duff, Mathew Hurley, Adéla Nacer, Paul W. Bowyer, Fidel Zavala, Azza H. Idris, Joseph R. Francica, Robert A. Seder, and Rogerio Amino

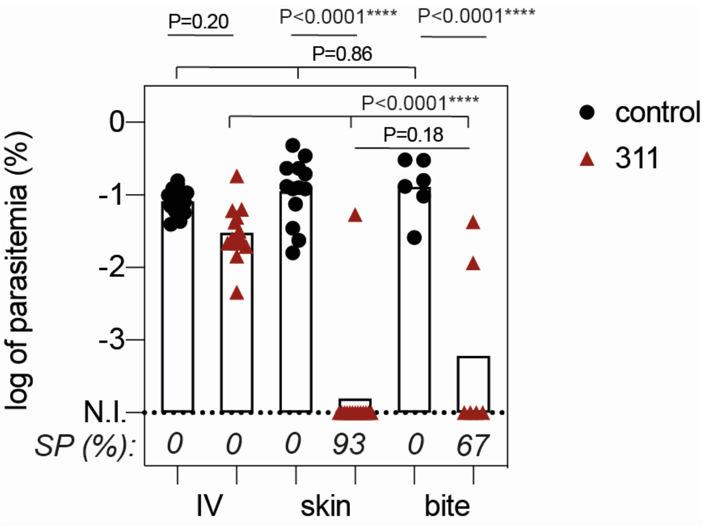

Figure S1. Protection after mosquito bite challenge is more similar to skin challenge than IV challenge. Related to Figure 1. Comparison of the parasitemia at day 5 post-challenge in PBS control or 311-immunized animals challenged with 1,000 PbPf sporozoites IV (n=13 mice, data from Fig. 1), 5,000 PbPf sporozoites in the skin (n=13 mice, data from Fig. 1), or 10 infected mosquitos for 10 minutes (n=6 mice). Percentage of sterilely protected (SP) mice is depicted below the bars. Statistical significance was determined by one-way ANOVA with Holm-Sidak correction for multiple comparisons (****, P<0.0001). N.I.: non-infected.

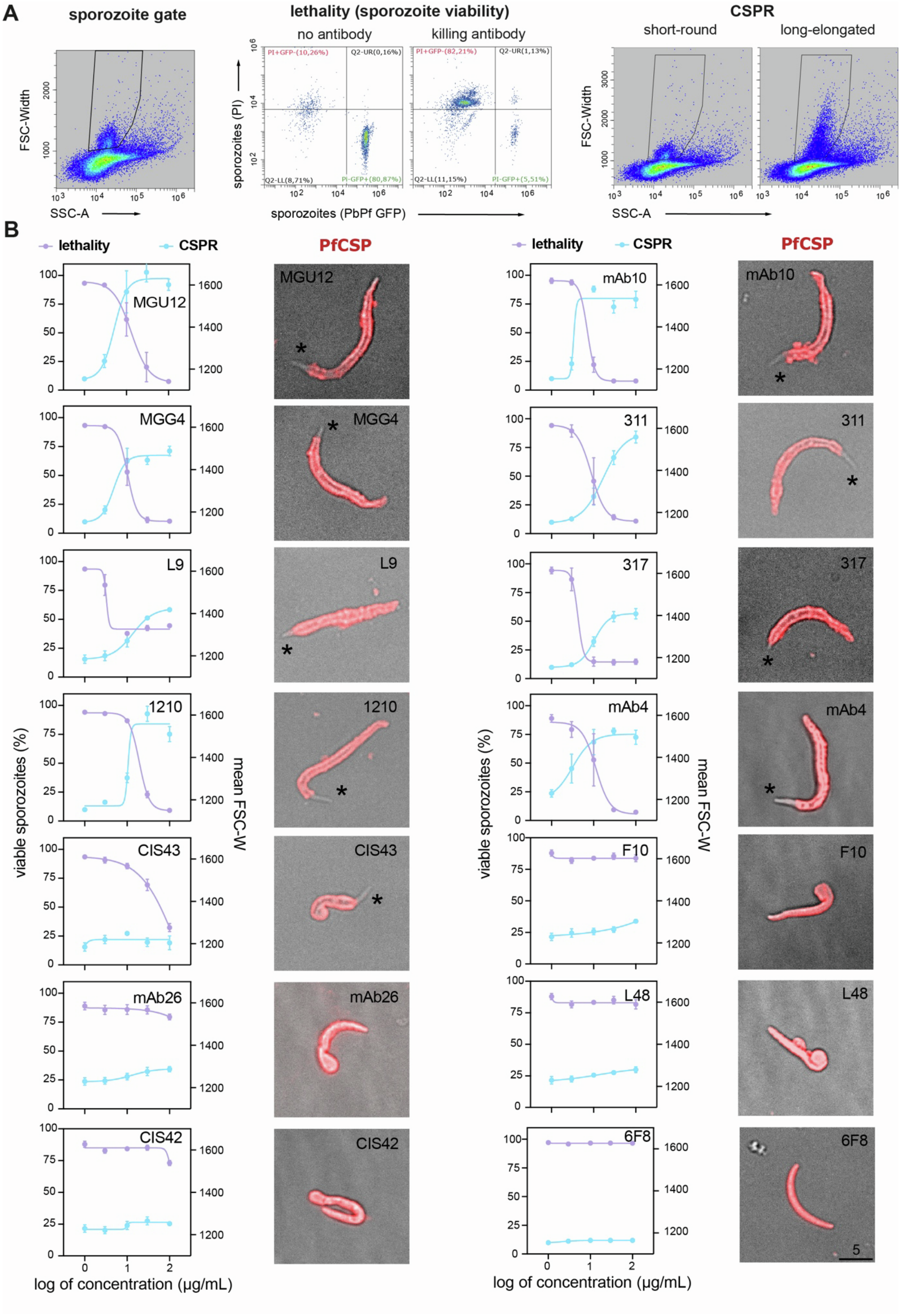
A
sporozoite gate
lethality (sporozoite viability)
no antibody
killing antibody
CSPR
short-round
long-elongated
FSC-Width
SSC-A
sporozoites (PI)
sporozoites (PbPf GFP)
B
lethality
CSPR
PfCSP
viable sporozoites (%)
mean FSC-W
log of concentration (µg/mL)
MGU12
MGG4
L9
1210
CIS43
mAb26
CIS42
mAb10
311
317
mAb4
F10
L48
6F8

Figure S2. ***In vitro*** **cytotoxicity and CSPR activities of thirteen PfCSP hmAbs against sporozoites in suspension. Related to Figure 2. (A)** Representative flow cytometry pseudo-colored plots depicting the gating strategy used to measure sporozoite viability (cytotoxicity) and CSPR length in PbPf GFP sporozoites after incubation with no antibody or cytotoxic hmAb for 45 min at 37 °C. Viability was defined as the percentage of sporozoites that retained GFP fluorescence (green) and did not uptake propidium iodide (PI, red). CSPR length was determined by the mean of the forward scatter-width signal (FSC-W). **(B) Left:** graphs showing the sporozoite viability (hmAb cytotoxicity, purple) and CSPR length (blue) after incubation of PbPf sporozoites with the indicated hmAb concentrations for 45 min at 37°C. **Right:** representative microscopy pictures showing distinct CSPR patterns induced in PbPf sporozoites after incubation with 100 µg/mL hmAb for 45 min at 37ºC. PfCSP (red) was revealed by incubating sporozoites with Alexa Fluor™ 647-conjugated anti-human IgG secondary antibody. Asterisks indicate the unlabeled anterior pole of dead sporozoites (n=3-6 independent experiments, data are represented as mean ± SEM). Bar, 5 µm.

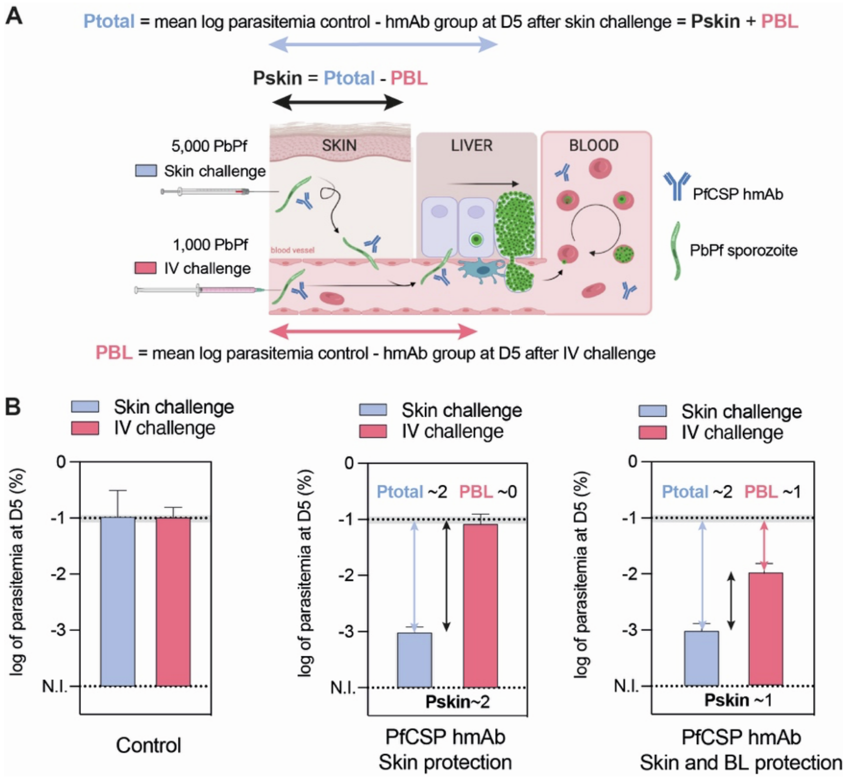

Figure S3. Quantification of the protection due to neutralization of sporozoites in the skin and in the blood or liver. Related to Figures 1 and 2. (A) To quantify protection in the main tissues where sporozoites transit, mice were challenged with 5,000 PbPf sporozoites microinjected in the skin (skin challenge, blue) or with 1,000 PbPf sporozoites inoculated in the blood to bypass the skin (IV challenge, red). Total protection (Ptotal, blue arrow) represents qualitatively, the neutralization of sporozoites in the skin, and blood and liver (BL) and quantitatively, the order of magnitude in parasitemia reduction after skin challenge. Ptotal is calculated as the mean log of parasitemia of control minus the mean log of hmAb passively immunized groups at D5. Protection in BL (PBL, red arrow) represents qualitatively, the neutralization of sporozoites in the blood and liver (BL) and quantitatively, the order of magnitude in parasitemia reduction after IV challenge. PBL is calculated as the mean log of parasitemia of control minus the mean log of hmAb passively immunized groups at D5. Protection in the skin (Pskin, black arrow) represents qualitatively, the neutralization of sporozoites in the skin and quantitatively the order of magnitude in parasitemia reduction due to sporozoite transit through the skin. Pskin is calculated as Ptotal minus PBL. Cartoon created using Biorender. **(B) left panel:** Skin (blue) and IV (red) challenges lead to similar parasitemia in control mice, indicating the same level of liver infection. **middle panel:** Example of a skin-protective hmAb showing ~100-fold decrease in parasitemia after skin challenge and no decrease after IV challenge (Ptotal=~2, PBL=~0, Pskin=~2). **right panel:** Example of a skin and BL-protective hmAb showing ~10-fold decrease in parasitemia after skin and after IV challenge (Ptotal=~2, PBL=~1, Pskin=~1). Cartoon created with BioRender.com.

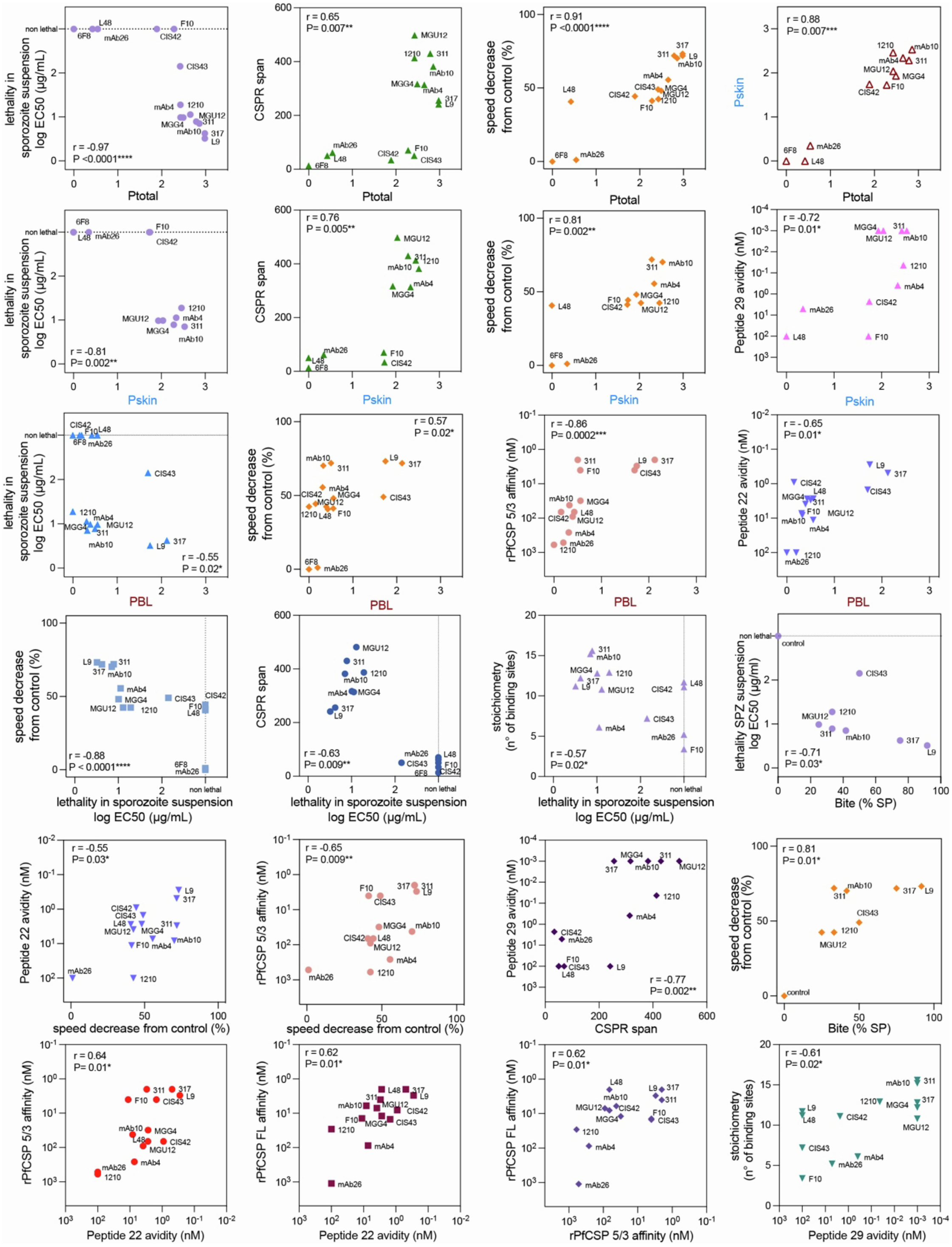

Figure S4. Correlation between the *in vitro* and *in vivo* properties of the PfCSP hmAb panel. Related to Figure 2. Plots depicting significant correlations (P<0.05) shown in the correlation matrix of Figure 2F. The Spearman correlation coefficient r and P-value for each correlation are depicted in each respective graph. CSPR span is the top minus the bottom of the sigmoidal FSC-W curve.

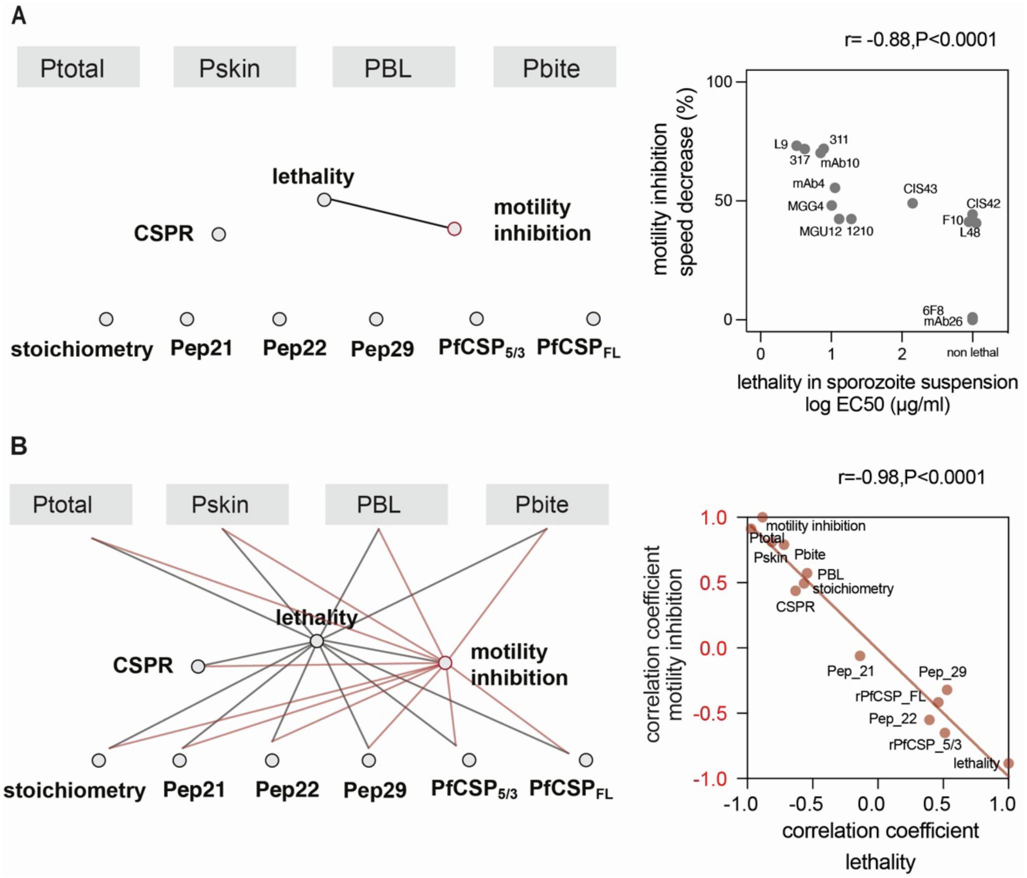

Figure S5. Strong correlation between lethality and motility inhibition. Related to Figure 2. A) Left: Diagram shows *in vivo* protection (Ptotal, Pskin, PBL, Pbite), and *in vitro* neutralization and binding parameters measured for the hmAb panel, emphasizing the relationship between lethality and motility inhibition. **Right:** The graph shows the strong Spearman's correlation between lethality and motility inhibition (r= -0.88, P<0.0001). **B) Left:** Diagram shows 4 *in vivo* and 9 *in vitro* parameters measured using the hmAb panel. Lines show the 12 parameters tested for correlation with lethality (black) and motility inhibition (red). For each pair of parameters, a coefficient of correlation (r) measuring the level of association is calculated as shown in (A). **Right:** The graph illustrates nearly perfect Pearson's correlation between the correlation coefficients of lethality and motility inhibition, showing their very strong dependence (r= -0.98, P<0.0001).

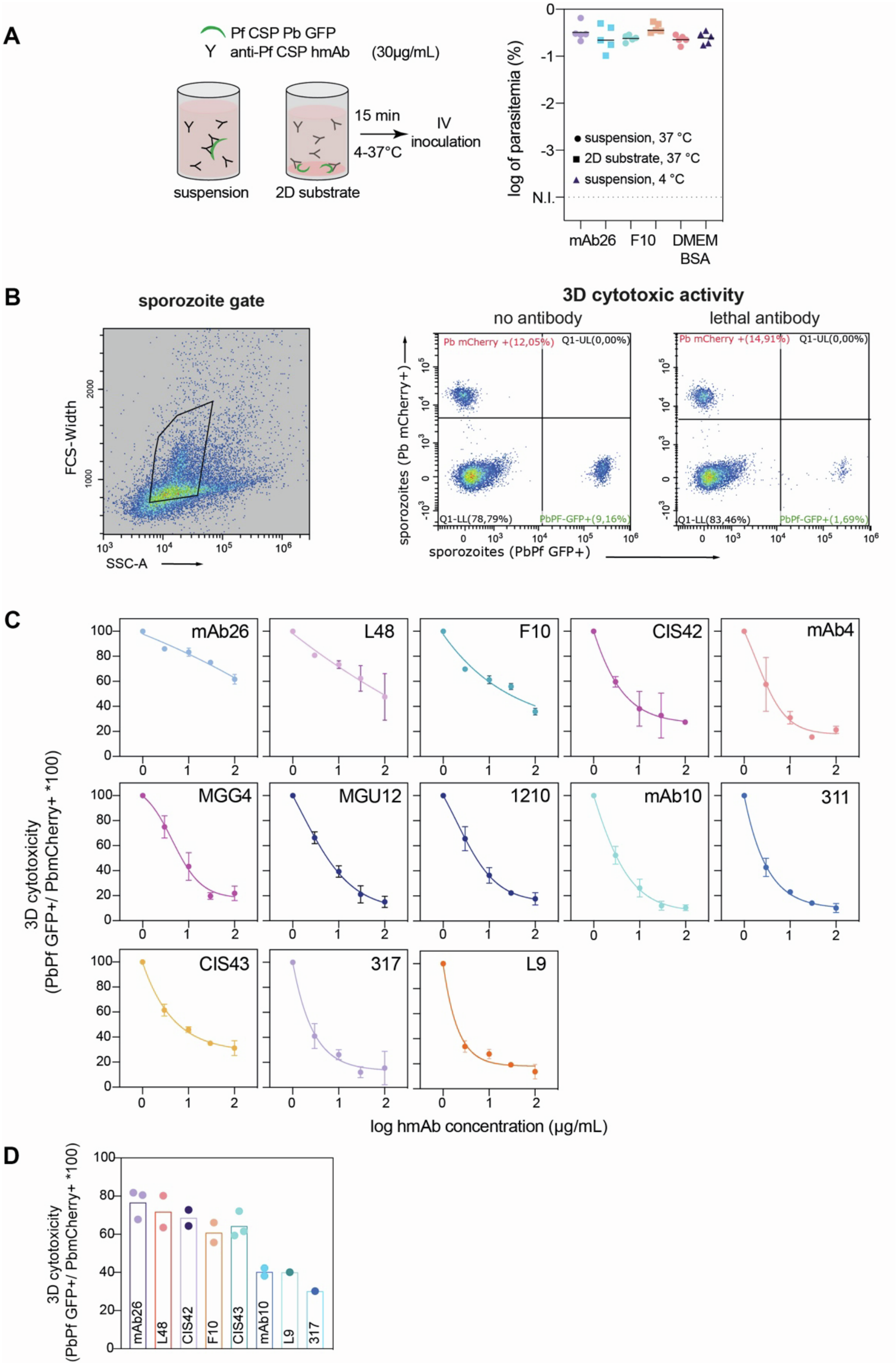

Figure S6. Development of *in vitro* assays to measure the impact of hmAbs on sporozoite infectivity. Related to Figure 4. (A) Left: Scheme for preincubating PbPf sporozoites with 30 µg/mL of hmAbs for 15 min at 4-37ºC in suspension or on a 2D substrate prior to IV challenge. **Right:** log parasitemia 5 days after IV challenge. As control, sporozoites were incubated in 0.5%

BSA/DMEM without hmAb. **(B)** Gating strategy of the 3D cytotoxicity assay to assess the viability of PbPf GFP$^+$ sporozoites and Pb mCherry$^+$ sporozoites after incubation with or without cytotoxic hmAb for 45 min at 37°C in 80% Matrigel. Pb mCherry$^+$ sporozoites were used to control sporozoite recovery after Matrigel depolymerization and to determine the viability of the hmAb susceptible PbPf GFP$^+$ sporozoites. **(C)** 3D cytotoxicity after incubation with varying hmAb concentrations in Matrigel (n=3 independent experiments, are represented as mean ± SEM). **(D)** Percentage of recovered PbPf GFP$^+$ sporozoites compared to Pb mCherry$^+$ sporozoites after incubation with 30 µg/mL hmAb in 80% Matrigel for 15 min prior to inoculation of sporozoites into mice (Fig. 4C).
